# Precise Regulation Strategy for Fluorescence Wavelength of Aggregation‐Induced Emission Carbon Dots

**DOI:** 10.1002/advs.202409345

**Published:** 2024-11-03

**Authors:** Liu Ding, Xilang Jin, Yuchong Gao, Shouwang Kang, Haiyan Bai, Xuehao Ma, Taotao Ai, Hongwei Zhou, Weixing Chen

**Affiliations:** ^1^ Engineering Research Center of Light Stabilizers for Polymer Materials Universities of Shaanxi Province School of Materials and Chemical Engineering Xi'an Technological University Xi'an 710021 P. R. China; ^2^ School of Material Science and Engineering Shaanxi University of Technology Hanzhong 723000 P. R. China; ^3^ Department of Materials Science and Engineering University of Pennsylvania Philadelphia PA 19104 USA

**Keywords:** aggregation‐induced emission (AIE), graphite nitrogen doping, multi‐color emission, multi‐information encryption, sp^2^ domains

## Abstract

Aggregation‐induced emission (AIE) carbon dot (CDs) in solid state with tunable multicolor emissions have sparked significant interest in multidimensional anti‐counterfeiting. However, the realization of solid‐state fluorescence (SSF) by AIE effect and the regulation of fluorescence wavelength in solid state is a great challenge. In order to solve this dilemma, the AIE method to prepare multi‐color solid‐state CDs with fluorescence wavelengths ranging from bright blue to red emission is employed. Specifically, by using thiosalicylic acid and carbonyl hydrazine as precursors, the fluorescence wavelength can be accurately adjusted by varying the reaction temperature from 150 to 230 °C or changing the molar ratio of the precursors from 1:1 to 1:2. Structural analysis and theoretical calculations consistently indicate that increasing the sp^2^ domains or doping with graphite nitrogen both cause a redshift in the fluorescence wavelength of CDs in the solid state. Moreover, with the multi‐dimensional and adjustable fluorescence wavelength, the application of AIE CDs in the fields of multi‐anti‐counterfeiting encryption, ink printing, and screen printing is demonstrated. All in all, this work opens up a new way for preparing solid‐state multi‐color CDs using AIE effect, and further proposes an innovative strategy for controlling fluorescence wavelengths.

## Graphical abstract

Herein, they proposed an efficient and precise wavelength tuning mechanism for the Aggregation‐induced emission (AIE) carbon dots (CDs). Specifically, by using thiosalicylic acid and carbonyl hydrazine as precursors, they can accurately adjust the fluorescence wavelength by varying the reaction temperature or changing the molar ratio of the precursors. Moreover, the application of AIE CDs in the fields of multi anti‐counterfeiting encryption has been demonstrated.

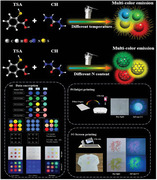

## 1 Introduction

Carbon dots (CDs) represent a novel class carbon‐based nanomaterials that possess tunable photoluminescence (PL), good solubility, high quantum yield (QY), and diverse synthetic methods.^[^ ^1^ ^]^ These intrinsic advantages make CDs an attractive candidate for applications in optoelectronic devices^[^ ^2^ ^]^ and multi‐anticounterfeiting.^[^ ^3^ ^]^ However, the aggregation‐caused quenching (ACQ) of the solid‐state fluorescence (SSF) of CDs greatly limits the construction of CDs‐based high‐efficiency illumination and display devices. The opposite of the ACQ effect, the aggregation‐induced emission (AIE) phenomenon constitutes an efficient strategy for the synthesis of CDs with SSF.^[^ ^4^ ^]^ The resultant AIE‐active CDs are capable of self‐quenching in the solid state without the need for additional treatment and can rapidly precipitate into powder upon the addition of inferior solvents.^[^ ^5^ ^]^ These distinctive attributes not only contribute to maximizing the SSF efficiency of CDs but also enhance their yield, thereby endowing the AIE technology with tremendous potential in the scalable production of CDs with a single‐step high‐efficiency SSF. To a significant extent, the AIE effect remains an underexplored tactic for enhancing the SSF efficiency of CDs.^[^ ^6^ ^]^

The CDs with tunable multicolor emissions in the solid state can also grant the CDs more flexible applications in many fields. Nowadays, most researchers agree that graphitization and surface functionalization are the two important factors affecting the emissive color of SSF CDs.^[^ ^7^ ^]^ Recently, multi‐color CDs can be harvested just by choosing different precursors^[^ ^8^ ^]^ and controlling reaction conditions such as the ratio of reactants,^[^ ^9^ ^]^ solvents,^[^ ^10^ ^]^ temperature,^[^ ^11^ ^]^ and pH.^[^ ^12^ ^]^ Wang and coworkers^[^ ^13^ ^]^ synthesized CDs by regulating reactant ratio and microwave power, the increase in both power and ratio of PG/urea synergistically leads to large conjugated sp^2^ size and high graphitization degree of CDs. Hu and his coworkers^[^ ^14^ ^]^ achieved multicolor emission CDs by adjusting the type and amount of nitrogenous precursors to control the content of graphitic nitrogen in carbon cores, thus realizing efficient full‐color emission. Ding and his coworkers^[^ ^15^ ^]^ used AIE strategy to prepare full‐color SSF CDs by adjusting the content of precursors and reaction time through one‐step microwave‐assisted pyrolysis. Our previous research^[^ ^16^ ^]^ also has succeeded in the preparation of AIE CDs with tunable multicolor emissions by using selected precursors, different content of precursors, and different reaction temperatures. However, these studies achieved multicolor CDs by adjusting two or more parameters simultaneously to control the fluorescence wavelengths, resulting in the analysis of the regulation mechanism being more complex.

Based on the above, a facile and efficient synthesis of multi‐color AIE CDs by controlling the single‐factor reaction temperature or the content of nitrogenous precursor was realized. Employing a fixed molar ratio of thiosalicylic acid (TSA) and carbonyl hydrazine (CH) as precursors, blue, green, yellow, and red emission AIE CDs were obtained with different reaction temperatures. Interestingly, under the same reaction conditions, only a slight adjustment of the molar ratio of the precursors can obtain green, yellow, and red‐emissive CDs. A combination of the structural analysis and theoretical calculations was performed to reveal that the effect of the increase of sp^2^ domains or graphite N doping on the fluorescence wavelength redshift was studied. Furthermore, based on the optical properties, the multi‐color AIE CDs can be used for multiple anticounterfeiting information encryption.

## 2 Results and Discussion

### 2.1 Design and Preparation of T‐CCDs

**Scheme 1:**
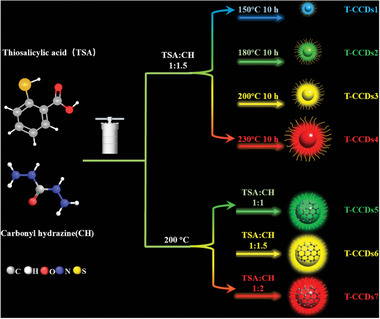
Illustration of T‐CCDs formation.

All of the CDs were obtained through solvothermal reaction with the thiosalicylic acid and carbonyl hydrazine as the precursors. A series of SSF CDs with different fluorescence colors were obtained by adjusting reaction temperature and molar ratio of precursor simultaneously (Figure S1, Supporting Information). In order to more accurately judge the regulation of fluorescence wavelength by a single variable, only one factor of the reaction temperature or the molar ratio of reactants was controlled. By varying the reaction temperature of the reaction from 150, 180, 200 to 230 °C, a series of SSF CDs with emission maxima ranging from blue to red under 365 nm excitation were obtained. For convenience, these samples were labeled as T‐CCDs1, T‐CCDs2, T‐CCDs3, and T‐CCDs4, respectively. For changing the content of nitrogen‐containing precursors, a series of experiments were conducted at different reaction temperatures, and the trend of fluorescence redshift induced by increasing nitrogen content was shown in each system by comparison (Figure S2, Supporting Information). In order to present the color more vividly, a reaction system with a reaction temperature of 200 °C was selected. Finally, the reaction temperature and reaction time were kept at 200 °C for 10 h, the molar ratio of TSA and CH was adjusted from 1:1 to 1:1.5 and 1:2, and a series of green, yellow, and red CDs were obtained, labeled as T‐CCDs5, T‐CCDs6, and T‐CCDs7, respectively. The synthetic routes and the colors of CDs were illustrated in **Scheme** **1**.

### 2.2 Characterization of T‐CCDs

**Figure 1:**
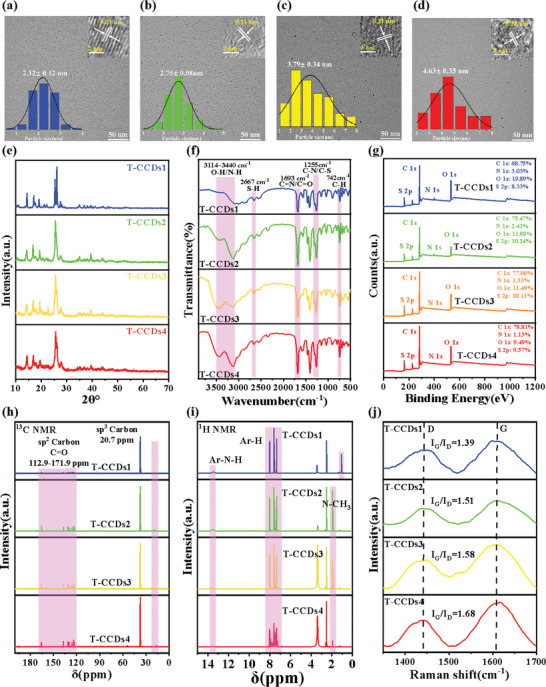
a–d) TEM image and the size distribution of T‐CCDs. e) XRD image of T‐CCDs. f) FT‐IR spectra of T‐CCDs g). XPS spectra of T‐CCDs. h) ^13^C NMR of T‐CCDs. i) ^1^H NMR spectra of T‐CCDs. j) Raman spectra of T‐CCDs.

A detailed analysis was conducted on the morphology and chemical structure of T‐CCDs. The particle size of T‐CCDs was observed using transmission electron microscopy (TEM) technology, with the specific results illustrated in **Figure** **1**a–d. The findings revealed that the T‐CCDs exhibited excellent uniformity and dispersity in size, with average particle diameters of 2.12 nm (T‐CCDs1), 2.75 nm (T‐CCDs2), 3.79 nm (T‐CCDs3), and 4.63 nm (T‐CCDs4), respectively. Additionally, the lattice fringes of T‐CCDs displayed an interplanar spacing of ≈0.21 nm, which corresponded to the (100) lattice plane of graphite. Concurrently, the presence of water led to the observation of some black aggregates of CDs, as shown in Figure S3 (Supporting Information), indicating the aggregation behavior of T‐CCDs. The X‐ray diffraction (XRD) pattern of the T‐CCDs (Figure 1e) exhibited a prominent peak at ≈26°, which corresponds to the (002) inter‐planar spacing of graphite.^[^ ^17^ ^]^ Overall, the XRD patterns corroborated the findings from TEM and offered deeper insights into the microstructural characteristics of the synthesized CDs.

The Fourier transform infrared spectra (FT‐IR) of T‐CCDs1‐4 (Figure 1f) showed multiple absorption bands at 3114 cm^−1^ corresponding to the stretching vibrations of O─H/N─H on the CDs surface. The absorption bands of 2667 and 1693 cm^−1^ were attributed to S─H and C═O/C═N stretches, respectively, and the band at 1423 cm^−1^ was assigned to C─N═C stretching.^[^ ^18^ ^]^ The 1255 cm^−1^ band in the FT‐IR spectrum corresponds to the stretching vibrations of C─N/C─S bonds.^[^ ^14^ ^]^ The full spectrum of X‐ray photoelectron spectroscopy (XPS) shows that all T‐CCDs are composed of C, O, N, and S elements (Figure 1g). In the high‐resolution C 1s XPS spectrum (Figure S4a–d and Table S1, Supporting Information), with the red shift of CDs emission, the sp^2^ hybrid carbon atoms (C═C/C─C) at 283.8 eV gradually increase. The O 1s XPS spectra show that in CD, 531.2 and 532.58 eV correspond to C═O and C─OH/C─O─C, respectively.^[^ ^9^ ^]^ The relative amount of sp^2^ carbon (C─O) bond increases with the red‐shift emission of CDs, indicating the size of sp^2^‐conjugated domains in the CDs increases after the dehydration reaction. It can be observed in the N 1s spectrum that the three component peaks at 399.2, 400.1, and 400.5 eV in the N 1s band represent pyrrolic N, pyridinic N, and graphitic N, respectively. The S 2p XPS spectrum shows that 162.8 eV C─S and 164.1 eV S─H are related.^[^ ^19^ ^]^ In addition, the results of XPS are completely consistent with the FT‐IR data.

To substantiate the proposed structure of T‐CCDs, spectral measurements via ^1^H NMR and ^13^C NMR were conducted. Given the distinct chemical environments of sp^3^ and sp^2^ carbons, the ^13^C NMR spectrum (as depicted in Figure 1h) exhibited well‐discriminated signals with chemical shifts (𝛿) ranging from 8 to 80 ppm and 90 to 180 ppm, respectively. The ^13^C NMR spectra of the T‐CCDs revealed that the chemical shift from 112.9 to 172.9 was attributed to aromatic sp^2^ carbons, and its relative content increases with the red shift emission of CDs. In the ^1^H NMR spectrum (Figure 1i) of T‐CCDs (d_6_‐DMSO as the solvent), a triplet peak at 𝛿≈2 corresponding to N─CH_3_ was observed. The aromatic hydrogen (7.0≈8.0) showed clear differentiation in the ^1^H NMR spectra.^[^ ^7a^ ^]^

Figure 1j displays the Raman spectra of the four types of CDs, wherein the two prominent peaks at 1451 and 1608 cm^−1^ correspond to the characteristics of disordered sp^3^ carbon (D band) and the graphite carbon structure (G band), respectively.^[^ ^7b^ ^]^ The intensity ratio of the G band to the D band (I_G_/I_D_) increases from 1.39 to 1.68, indicating an enhancement in the graphitization degree of the CDs as the emission spectra red‐shift. In the Mass Spectrometry (MS) spectra (Figure S5, Supporting Information), the value of m/z increases continuously from 290.1116 in T‐CCDs1, 328.9898 in T‐CCDs2, 459.0009 in T‐CCDs3, and 611.0189 in T‐CCDs4. Combined with Raman and MS analyses, it is suggested that increasing graphitization in the carbon core of the CDs^[^ ^20^ ^]^ can be realized by increasing the reaction temperature.^[^ ^21^ ^]^ The integrated analysis of Raman spectroscopy and mass spectrometry results leads to the conclusion that an increase in the reaction temperature facilitates the enhancement of the graphitization level of the carbon cores in CDs. At lower solvothermal temperatures, small‐sized sp^2^ domains are formed through a gradual carbonization process. As the solvothermal treatment temperature increases, the degree of carbonization progressively intensifies. This transformation is initially manifested by an increase in the number of graphitic conjugated fragments within the carbon cores, which is subsequently followed by the expansion of the aromatic and sp^2^ domains.^[^ ^7a^ ^]^

### 2.3 Fluorescence Properties and Its Regulatory Mechanism

**Figure 2:**
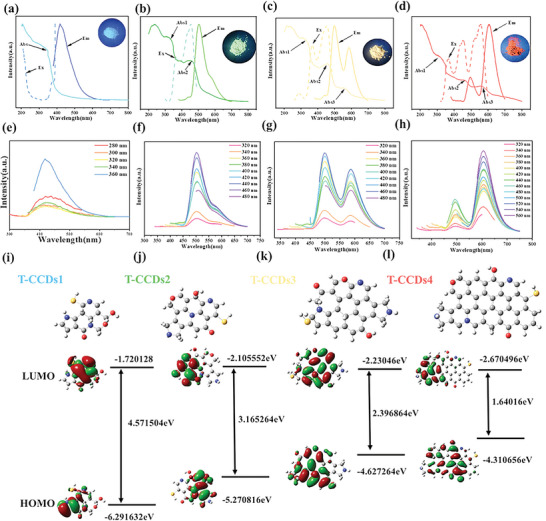
–d) UV–vis absorption, FL excitation (Ex), and emission (Em) of as‐prepared T‐CCDs 1–4 powder. e–h) Fluorescence emission of T‐CCDs1‐4 powder under different excitation wavelengths. i–l) Structural models and the calculation of LUMO and HUMO for T‐CCDs1‐4.

An investigation was conducted into the UV–vis absorption, photoluminescence (PL) excitation, and emission characteristics of T‐CCDs1‐4 powders. The UV–vis spectra (**Figures** **2**a‐d) reveal that the Abs‐1 peaks for T‐CCDs1, T‐CCDs2, T‐CCDs3, and T‐CCDs4 are within the range of 250–350 nm, while the corresponding Abs‐2 peaks are located at ≈300–350, 460, 460, and 560 nm, respectively. The Abs‐1 peaks correspond to the *π*–*π* ^*^ electronic transitions of C═C bonds within the carbonaceous cores.^[^ ^22^ ^]^ Additionally, the Abs‐2 peaks are consistent with the n‐*π* ^*^ electronic transitions associated with the surface states containing C═N/C═O, C─O, and C─S structures.^[^ ^23^ ^]^ To evaluate the optimal optical features of the T‐CCDs, PL analyses were conducted (Figure 2e‐h). Surprisingly, the T‐CCDs1, T‐CCDs2, T‐CCDs3, and T‐CCDs4 powders displayed blue, green, yellow, and red PL emissions, as shown in Figure 2a‐d. The PL spectra of T‐CCDs1 and T‐CCDs2 exhibit single fluorescence emission peaks at 410 and 500 nm and exhibit excitation‐independent behavior. T‐CCDs3 and T‐CCDs4 show noteworthy dual‐peak emissions, specifically, the dual emission peaks were located at 500 and 600 nm. Emission at short wavelengths primarily originates from the intrinsic states of the carbon cores in CDs, whereas emission at long wavelengths is mainly induced by the surface defects and functional groups on the CDs.^[^ ^24^ ^]^ The conjugated structure of the carbon source plays a crucial role in the preparation of CDs with double emission.^[^ ^25^ ^]^.

The types of chemical bonds and the distribution of functional groups on the surface of T‐CCDs were inferred through XPS and FT‐IR spectra. Concurrently, detailed analysis of the structural fragments within CDs was conducted using mass spectra. In conjunction with the previously mentioned Raman data, it was observed that among the four types of CDs, the carbon core of T‐CCDs4 exhibits the highest degree of graphitization, implying the presence of a more extensive π‐conjugated system. As shown in Figure 2i–l, these four rational structures of T‐CCDs were further verified using DFT calculations. At the B3LYP/6311G (d, p) basis set level, the calculations were subjected to symmetry optimization using the Gaussian 09 software package. Subsequently, the energies of the lowest unoccupied molecular orbital (LUMO) and the highest occupied molecular orbital (HOMO) were calculated, and the bandgap (Eg) was determined by the energy difference between the LUMO and HOMO. As the conjugated domain expanded, the LUMO energy slightly decreased, while the HOMO energy significantly increased, leading to a narrowing of the bandgap and a redshift in the emission wavelength. Significantly, the Eg values of four models closely correlated with the experimental data derived from UV–Vis absorption spectra and fluorescence spectra calculations (Figures S6 and S7, Supporting Information). The variation pattern of the Eg value obtained from the experiment is in alignment with the theoretical predictions, thereby substantiating the significant influence of the size effect during graphitization on the fluorescence emission of T‐CCDs.

### 2.4 Regulation of Fluorescence Wavelength by Nitrogen Doping

**Figure 3:**
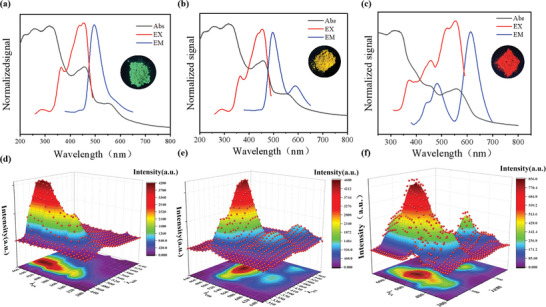
a–c) UV–vis absorption, FL excitation (Ex), and emission (Em) of as‐prepared T‐CCDs powder. d–f) The 3D contour fluorescence emission spectra of T‐CCDs. T‐CCDs5 (a,d) T‐CCDs6 (b,e), and T‐CCDs7 (c,f).

In order to further analyze the other factors contributing to the redshift of fluorescence wavelength in solid state, both the reaction temperature and time were controlled stably. For tuning their emission color, the ratio of carbonyl hydrazine was changed to control the nitrogen content in CDs. The optical properties of the T‐CCDs5‐7 powder were evaluated by UV–Vis absorption spectroscopy and fluorescence spectra. The UV–vis spectra (**Figure** **3**a–c) reveals that T‐CCDs5‐7, similar to the T‐CCDs1‐4, also exhibit two absorption peaks corresponding to *π*–*π* ^*^ transitions of the carbonaceous core and n–*π* ^*^ transitions of the surface states.^[^ ^26^ ^]^ It can be seen that T‐CCDs5 exhibits green fluorescence emission in solid state, the excitation and emission were 450 and 500 nm. Distinct from T‐CCDs5, the fluorescence color of T‐CCDs6 is influenced not only by the strong emission center at 500 nm but also by the concurrent action of the weak emission center at 600 nm. Moreover, in the dual‐emission characteristics, the emission of yellow and green light can be achieved only by adjusting the emission intensity ≈600 nm. As for T‐CCDs7, the emission center at 600 nm is more prominent, whereas the emission center at 500 nm is relatively weaker, which showed the red emission under 365 nm UV. Interestingly, the higher intensity of emission at 600 nm caused the fluorescence wavelength to shift to red. The 3D fluorescence spectra of three CDs powder demonstrated that optimal excitation wavelengths for T‐CCDs5, T‐CCDs6, and T‐CCDs7 powders were 420, 430, and 500 nm, respectively. The optimal emission wavelengths for the three powders were 480, 500, and 600 nm.

**Figure 4:**
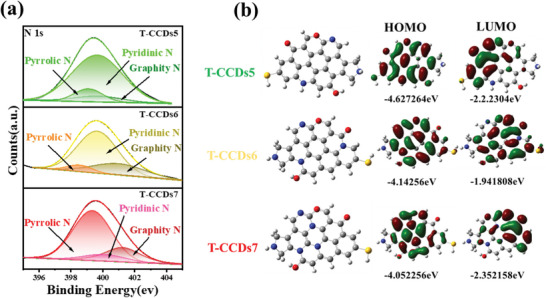
a) N1s XPS spectra of T‐CCDs5‐7. b) Structural models and the calculation of LUMO and HUMO for T‐CCDs5‐7.

However, the precise mechanism by which nitrogen influences the photoluminescent properties of CDs is not yet fully understood, as nitrogen can be incorporated into the CDs' structure in a variety of functional group forms.^[^ ^27^ ^]^ The structure of three CDs was proved by XPS, FT‐IR, MS spectra (Figures S8,  S9, and S12, Supporting Information), and DFT calculation. The atomic ratios of C, N, O, and S elements of T‐CCDs5, T‐CCDs6, and T‐CCDs7 were calculated and presented in Figure S8 (Supporting Information). Nitrogen content of T‐CCDs5, T‐CCDs6 and T‐CCDs7 is 1.56%, 2.97%, and 3.35%, respectively. As the nitrogen content increases, the emission characteristics of CDs gradually shift toward the red wavelength, which might represent another factor influencing the changes in their optical properties. The high‐resolution N 1s spectrum of three T‐CCDs are resolved into three peaks located at 398.4, 399.1, and 400.3 eV, related to pyrrolic N, pyridinic N, and graphitic N^[^ ^28^ ^]^ (**Figure** **4**a; Table S2, Supporting Information). From T‐CCDs5 to T‐CCDs7, it has been demonstrated that the graphite N increases significantly. At the same time, Raman spectra (Figure S14, Supporting Information) show that the degree of conjugation does not change significantly, so it shows that the increase of graphite N content may lead to the red shift of CDs.

After a series of structural characterization of these three CDs, the structural design and optimization were carried out according to the experimental results, and the UV–vis and fluorescence spectra were simulated according to their structures (Figure S15, Supporting Information). This study, through theoretical calculations and experimental validation, demonstrates that the three types of CDs products exhibit similar structures; however, due to variations in the types of nitrogen atoms and their positions within the structure, their fluorescence emission properties differ (Figure 4b). Remarkably, due to the improved content of graphitic N from T‐CCDs5 to T‐CCDs7, the CDs obtained red‐shift PL emission from green to red. The increase in graphitized nitrogen content within CDs not only enhances the probability of radiative processes by providing delocalized electrons but also reduces the likelihood of non‐radiative processes by augmenting the rigidity of the aromatic domains.^[^ ^21^ ^]^

### 2.5 Mechanism of AIE Effect

**Figure 5:**
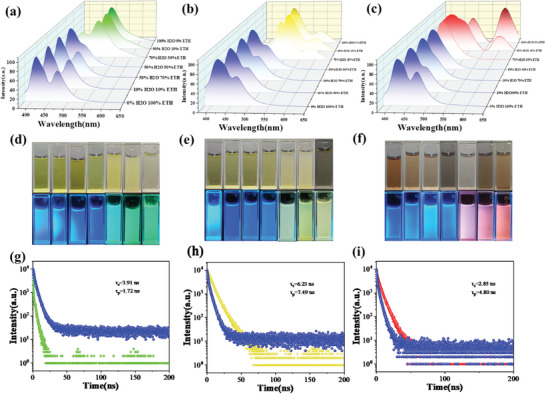
a–c) Fluorescence emission spectra of the T‐CCDs as‐prepared solution with varying volume ratio of water. d–f) photographs of the T‐CCDs as‐prepared solution with varying contents of water. g–i) Fluorescence lifetime decay of the T‐CCDs Powder and solution. a,d,g) T‐CCDs5, b,e,h) T‐CCDs6, and c,f,i) T‐CCDs7.

As mentioned above, the SSF of the T‐CCDs was achieved by overcoming the ACQ effect through AIE behavior. To further study the influence of aggregation on the fluorescence properties, the fluorescence spectra of T‐CCDs solution were recorded (Figure S16, Supporting Information). When the three CDs are uniformly dispersed in ethanol, they usually exhibit blue emission. And when they aggregated in water or in solid state, they exhibit their respective fluorescence characteristics (**Figure** **5**a–c). For the T‐CCDs5, T‐CCDs6, and T‐CCDs7, the fluorescence color changes from green to yellow, and red with the water adding, respectively. Under a 365 nm UV excitation (Figure 5d–f), the transparent liquids (volume ratio of water <50%) display blue fluorescence, while in 100% water, R‐CDs, Y‐CDs, and G‐CDs aggregate to form larger particles, with their fluorescence shifting toward red, yellow, and green, respectively, which further corroborates the AIE phenomenon of CDs in water. Upon the addition of a poor solvent (water), CDs commence to aggregate, thereby restricting intramolecular vibrations, resulting in the dissipation of energy primarily in the form of radiation.

An in‐depth investigation into the origins of the AIE effect in solids is a subject worthy of further study. The AIE properties of T‐CCDs may be due to the restriction of intramolecular motion (RIM) mechanism: in the dispersed state, the fluorescence molecules of T‐CCDs can vibrate freely, causing the excited state electrons to dissipate through non‐radiative transition channels. In highly concentrated CDs solutions or in the solid state of CDs, the hydrogen bonds formed between CDs molecules play a crucial role in enhancing the emission intensity associated with aggregation. Detailed analysis of the FT‐IR (Figure S9, Supporting Information) indicated the ─OH and ─NH groups formed intermolecular hydrogen bonds, causing the ─OH stretching modes to shift to lower wavenumber and a stabilization of the aggregation between CDs molecules.^[^ ^29^ ^]^ In addition, electron clouds on the *π* bond on the aromatic ring can provide lone pairs of electrons to form intermolecular hydrogen bonds with the surface ─OH. These two types of intermolecular hydrogen bonds (C─H ∙∙∙*𝜋* and O─H ∙∙∙N) result in stronger intermolecular forces and greater intermolecular distances for CDs in the aggregated state (Figure S10, Supporting Information). To verify the presence of hydrogen bonding in the aggregated state, the FT‐IR of CDs in the dispersed state and the aggregation state were measured (Figure S11, Supporting Information). Detailed analysis of the FT‐IR indicated the C─H ∙∙∙*𝜋* and O─H ∙∙∙N groups formed intermolecular hydrogen bonds, causing the O─H/N─H stretching modes to shift to lower wavenumber.^[^ ^30^ ^]^ The *𝜋*–*𝜋* interaction caused by direct contact between nanoparticles is reduced, and the intramolecular motion is limited, so that radiation transition is the main way for excited electrons to return to the ground state. CDs exhibits strong SSF.^[^ ^31^ ^]^ Moreover, the pyridinic N and graphitic N within the T‐CCD structure contribute to conjugated configurations, resulting in reduced nonradiative transitions, such as heat release from single bond rotations.^[^ ^32^ ^]^ Hence, the radiative transitions increased and the brightness were improved.

The time‐resolved PL decay (TRPL) curves of T‐CCDs5, T‐CCDs6, and T‐CCDs7 were measured at the excitation wavelength of the optimal excitation of them. The fluorescence decay for the three CDs was measured at maximum emission. Equations (1) and (2) were used to fit the fluorescence decay curve and calculate the average lifetimes (τ_avg_) of the fluorescence decay process:

(1) $$I\left(t\right)=A_{1}\;\exp\left(-\frac{\tau }{\tau_{1}}\right)+A_{2}\exp\left(-\frac{\tau }{\tau_{2}}\right)$$

(2) $$\tau_{avg}=\frac{A_{1}{\tau_{1}}^{2}+A_{2}{\tau_{2}}^{2}}{A_{1}\tau_{1}+A_{2}\tau_{2}}$$

where *τ*_1_ and *τ*_2_ refer to fluorescence decay lifetimes, *A*_1_ and *A*_2_ represent the pre‐exponential factors of *τ*_1_ and *τ*_2_, respectively. The statistical results are shown in **Table** **1**.

**Table 1:** QY, τ, K_r_, and K_nr_ of T‐CCDs5‐7 solution and powder excited by 360 nm.

	QY	τ (ns)	K_r_[ns^−1^]	K_nr_[ns^−1^]
**T‐CCDs5 Powder**	70.6%	1.72	0.407	0.174
**T‐CCDs5 Solution**	13.6%	3.91	0.035	0.221
**T‐CCDs6 Powder**	17.2%	7.49	0.230	0.111
**T‐CCDs6 Solution**	5.2%	6.23	0.008	0.152
**T‐CCDs7 Powder**	39.1%	4.80	0.281	0.127
**T‐CCDs7 Solution**	4.6%	2.85	0.161	0.335

As shown in Figure 5g–i, these decay curves exhibit similar double exponential lifetimes, which indicates that the luminescence processes of all samples are similar. Next, the absolute quantum yield of T‐CCDs5‐7 respectively is merely13.6%, 5.2%, and 4.6% in solution and far below that in the solid state. This is a typical phenomenon of aggregation‐induced enhancement emission. Then, the K_r_ (radiative rate constants) and K_nr_ (non‐radiative rate constants) of the three T‐CCDs powder and T‐CCDs solution were calculated according to the following equations:

(3) $$K_{r}=\frac{QY}{\tau_{\mathrm{avg}}}$$

(4) $$K_{\mathrm{nr}}=\frac{1-QY}{\tau_{\mathrm{avg}}}$$

Table 1 shows a slightly higher K_r_ and a significantly lower K_nr_ in the solid state. This is well in accordance with the AIE mechanism of RIM.^[^ ^5^ ^]^

## 3 Applications of Multi Color AIE CDs Materials

**Figure 6:**
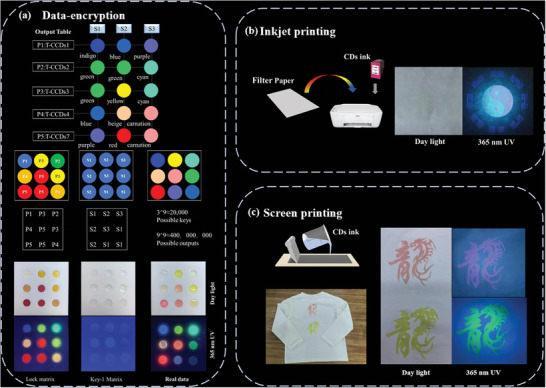
Application of T‐CCDs in multi‐information encryption.

Motivated by the remarkable optical properties of the T‐CCDs with tunable emission from blue to red, they have shown great potential in various applications. A solvent effect data‐encryption method was developed first. As shown in **Figure** **6**a, the 5×3 matrix containing five AIE CDs vertically, that is T‐CCDs1, T‐CCDs2, T‐CCDs3, T‐CCDs4, and T‐CCDs7, respectively, is defined as the “lock” matrix. There are three different solvents along the horizontal axis, that is, dimethyl sulfoxide (S1), dichloromethane (S2), and methylbenzene (S3), respectively, are defined as the “key” matrix. The 5×3 “output” matrix is generated by adding corresponding elements from both the “lock” and “key” matrices. When combining S1 with P1, P2, P3, P4, and P5, an indigo, green, green, blue, and purple output is generated, respectively. When combining S2 with P1, P2, P3, P4, and P5, a blue, green, yellow, beige, and red output is generated, respectively. A purple, cyan, cyan, carnation, and carnation are generated when combining the S3 with P1, P2, P3, P4, and P5. Therefore, only the “key” matrix containing three solvents in the correct positions will produce the desired “output” matrix. There are ≈20 000 possible “key” matrixes (i.e., 3ˆ9 = 19 683, where “3” indicates three solvents, and “9” represents nine positions in the matrix) and only one “key” matrix can decrypt the data. Considering nine different emission colors in the “output” matrix, ≈400 000 000 (9^9 = 387 420 489) wrong outputs but only one correct output could be generated. The above findings clearly illustrate the high level of data encryption security achieved by the proposed “lock‐key” strategy using a straightforward 5×3 matrix.

Additionally, these CDs exhibit outstanding chemical stability, making them highly suitable for rapid and intelligent fluorescent anti‐counterfeiting printing applications. By refilling the CDs into standard ink cartridges, they can be readily utilized in commercial desktop inkjet printers, such as the HP‐Deskjet 2132. As shown in Figure 6b, a traditional Chinese tai chi Eight diagrams printed with inkjet printing. Furthermore, the multicolor CDs can be doped in inks such as screen printing and printed out Chinese character “dragon” in visible light and showed fluorescent in 365 nm UV. Compared with the ink printing of traditional inkjet printers, screen printing has greater flexibility, just like the advertising slogan says, “Except air and water, printing everything”.

## 4 Conclusion

In summary, multi‐color AIE CDs were synthesized via the one‐step reaction with the same precursors. By manipulating the size of the sp^2^ conjugated domains through the adjustment of reaction temperature, the emission color of solid‐state T‐CCDs can be altered, transitioning from blue to red. Furthermore, with the increase in nitrogen doping concentration, the content of graphitized nitrogen within the carbon core also rises, thereby causing the emission color of the carbon core to shift from green to red. The fluorescence mechanism of T‐CCDs also revealed that the T‐CCDs exhibit AIE effect. The outstanding property of CDs opens exciting possibilities for applications in multi‐information encryption. Hence, this study not only offers a method based on the AIE effect for the synthesis of multicolored SSF CDs but also holds significant implications for the subsequent exploration of their mechanisms and practical application domains.

## 5 Experimental Section

Reagents, preparation, characterizations, and theoretical calculations are available in Supporting Information.

## Conflict of Interest

The authors declare no conflict of interest.

## Supporting information



Supporting Information

## Data Availability

The data that support the findings of this study are available in the supplementary material of this article.
