# Supplementary material for: Precise Regulation Strategy for Fluorescence Wavelength of Aggregation‐Induced Emission Carbon Dots
Source: Adv Sci (Weinh). 2024 Nov 3;11(48):2409345. doi: 10.1002/advs.202409345 (PMC11672272; doi:10.1002/advs.202409345)
Supplement: Supplementary file 1 — Supporting Information [file ADVS-11-2409345-s001.docx]

Supplementary Information

**Precise Regulation Strategy for Fluorescence Wavelength of Aggregation-Induced Emission Carbon Dots**

*Liu Ding, Xilang Jin*, Yuchong Gao, Shouwang Kang, Haiyan Bai, Xuehao Ma, Taotao Ai, Hongwei Zhou, Weixing Chen**

Materials

Reagent grade of mg Carbonyl hydrazine was bought from Anhui Zesheng Science Co., Ltd (Sichuan, China). Thiosalicylic acid and Salicylic acid were purchased from Sace Chemical Technology Co., LTD (Shanghai, China). Acetic acid, ethanol, normal hexane, dichloromethane, DMF, DMSO were purchased from TianLi Chemical Reagent Co., Ltd (Tianjin, China). All reagents were of analytical grade and used directly without further purification. Deionized (DI) water was used throughout this work.

Characterization

Transmission electron microscopy (TEM) observations were performed on a FEI Talos F200S. XPS were carried out with Thermo Scientific KAlpha+. Fourier transform infrared (FT-IR) spectra were obtained on a Nicolet 6700 FT-IR spectrometer. Fluorescence emission and excitation spectra were measured on a Hitachi F-4700 spectrophotometer at ambient conditions. Mass spectra were measured by electron spray ionization (ESI) using a Bruke rmicro TOF-QIIESI-Q-TOF LC/MS/MS spectrometer. NMR spectra (for ^1^H NMR at 400 MHz) were recorded on a Varian INOVA-400 MHz spectrometer using tetramethylsilane (TMS) as an internal standard. UV-Vis absorption spectra were recorded on a Cary 5000 UV-Vis spectra photometer. Fluorescence spectra and lifetime decay cures of samples were measured with HORIBA QuantaMater 8000 fluorescence spectrophotometer. Photographs/videos of fluorescence emission were taken using a HUAWEI cellphone (Mate 30) under excitation by a hand-hold UV lamp (365 nm).

**Synthesis of T-CCDs1-7**

Thiosalicylic acid (266 mg) and Carbonyl hydrazine (234 mg) (molar ratio 1:1.5) were dissolved into 40 mL acetic acid with ultrasonic treatment, then the solution was transferred into a 100 mL Teflon reactor and kept at 150 °C for 10 h in an air oven. The T-CCDs2, T-CCDs3, and T-CCDs4 were obtained with the reaction temperature at 180 °C, 200 °C, and 230 °C, respectively. After cooling down to room temperature naturally, the reaction solution was poured into 1 L boiled water and the liquid mixture was filtered through a 0.22 μm pore diameter microporous membrane. Then, the product dialyzed using a membrane (300 Da) for 48 h to remove any unreacted small molecules. Finally, the T-CCDs were obtained as a white powder by freeze-drying.

Thiosalicylic acid (315 mg) and Carbonyl hydrazine (185 mg) (molar ratio 1:1) were dissolved into 40 mL acetic acid with ultrasonic treatment, then the solution was transferred into a 100 mL Teflon reactor and kept at 200 °C for 10 h in an air oven The next processes were the same as those mentioned above and the T-CCDs5 was obtained. The T-CCDs6 and T-CCDs7 were obtained through the molar ratio at 1:1.5 and 1:2.

Preparation of anti-counterfeiting ink

(1) Preparation of Inkjet Printing Ink: CDs powder (0.1 g) and dissolve it in 5 mL of anhydrous ethanol. Ultrasonicate for 30 minutes to achieve uniform dispersion of CDs. Then, take 3 mL of deionized water and 2 mL of glycerol, add them to the above solution, and continue ultrasonication for 15 minutes. Finally, transfer the mixed solution into a clean inkjet printer cartridge.

(2) Preparation of Screen-Printing Ink: CDs powder (0.1 g) and dissolve it in 5 mL of commercially available transparent screen-printing ink. Ultrasonicate for 30 minutes to achieve uniform dispersion of CDs. Then, transfer 2 mL of the mixed ink into a customized screen-printing template and use a squeegee to coat the ink onto the fabric surface. Allow the ink to dry, and the fluorescent printing pattern will appear on the fabric.

Synthesis of CDs/PP Film

Mix T-CCDs powder (0.5 g) with polypropylene (PP) (3 g) thoroughly. Then, place the CDs-PP mixture into the hopper of an SCM blown film extrusion machine to produce plastic film. Set the barrel temperatures to 160 °C, 170 °C, and 175 °C, and the mold temperatures to 170 °C and 165 °C.

Computational Details

All calculations are performed with the Gaussian 09 program. The ground-state geometries of carbon dots were optimized by DFT B3LYP (Becke’s three-parameter hybrid function with the non-local correlation of Lee-Yang-Parr) functional, 6–311G(d) basis set (B3LYP/6-311G (d, p). The absorption spectra of carbon dots were calculated using the TDDFT method (at the B3LYP/6-311G (d, p) level) based on optimized ground-state geometries. The first excited state was optimized using the TDDFT method to calculate the emission energy (wavelength) which is the energy difference between the ground and the first excited state.

**
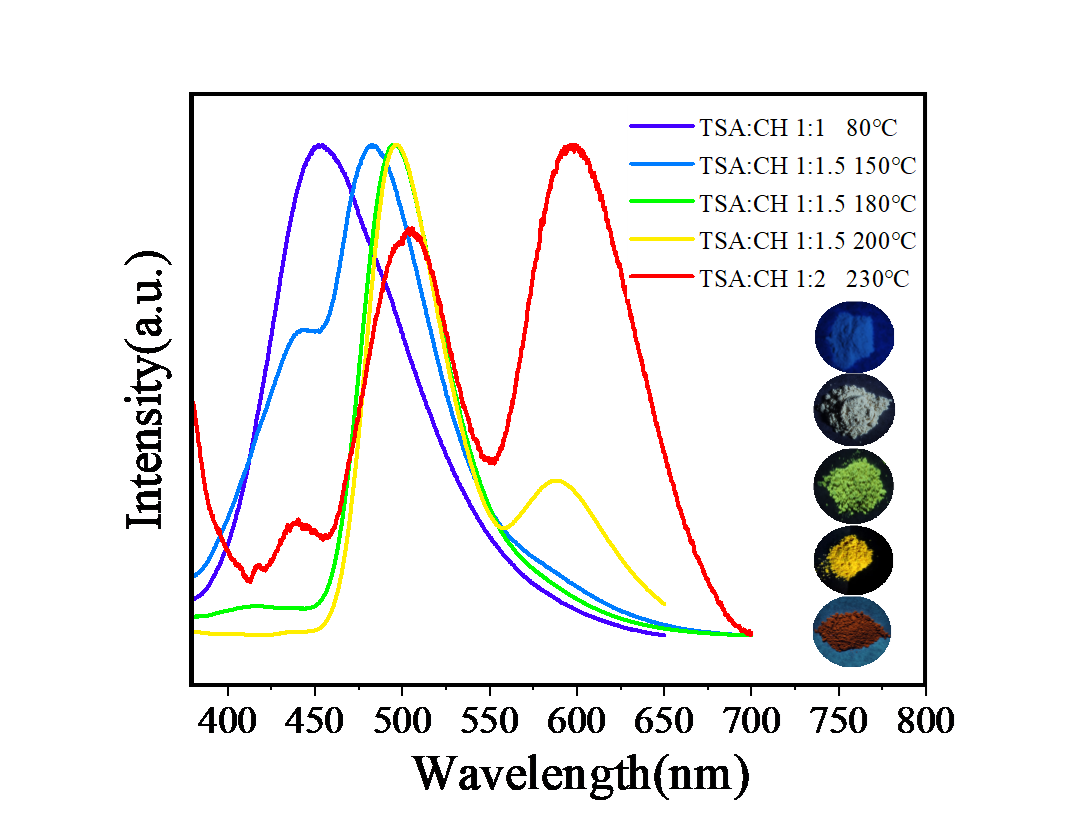
**

Figure S1. Multi-color CDs obtained under different reaction conditions


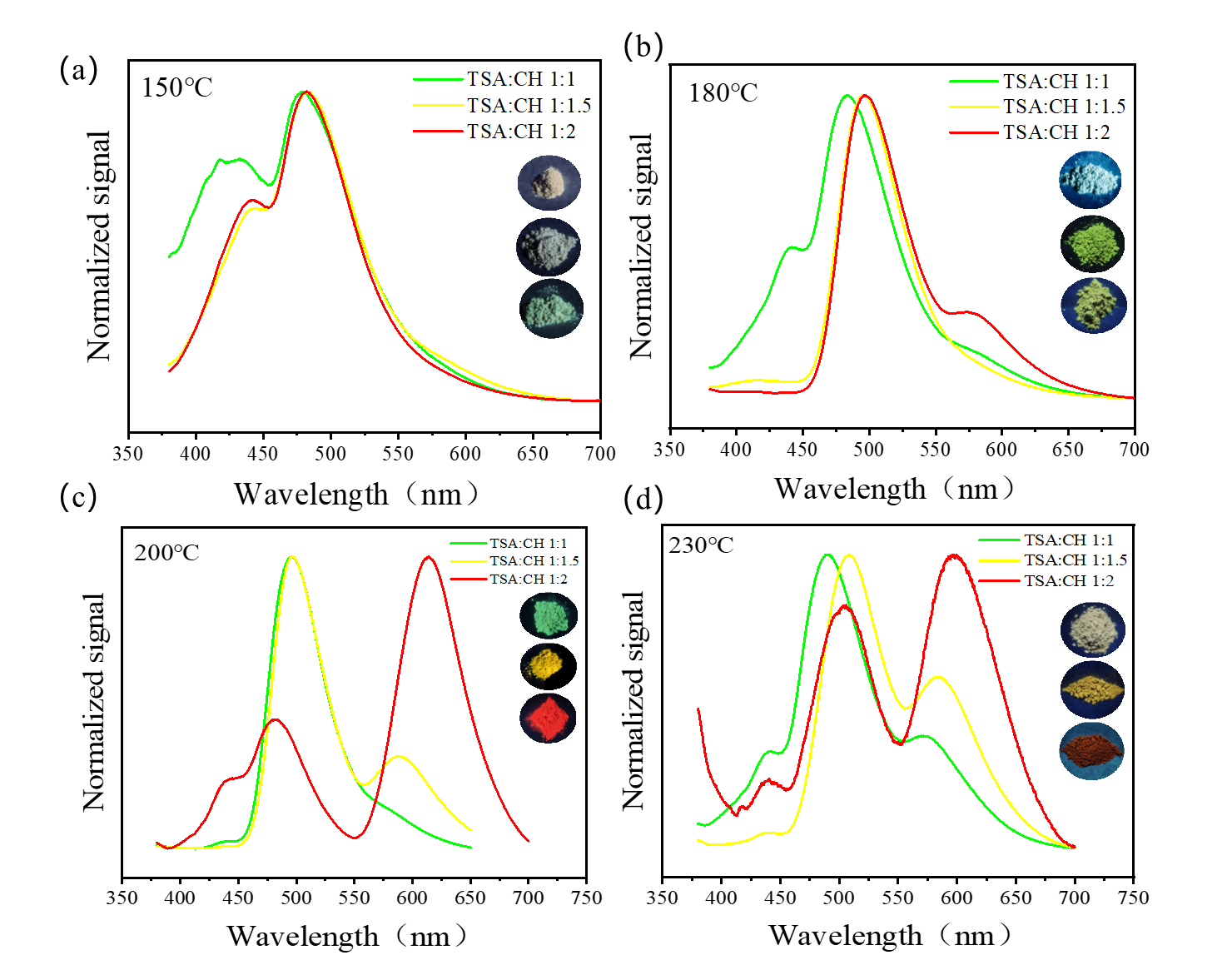


**Figure S2.** (a-d) PL spectra of T-CCDs with different molar ratio at different reaction temperature.

**
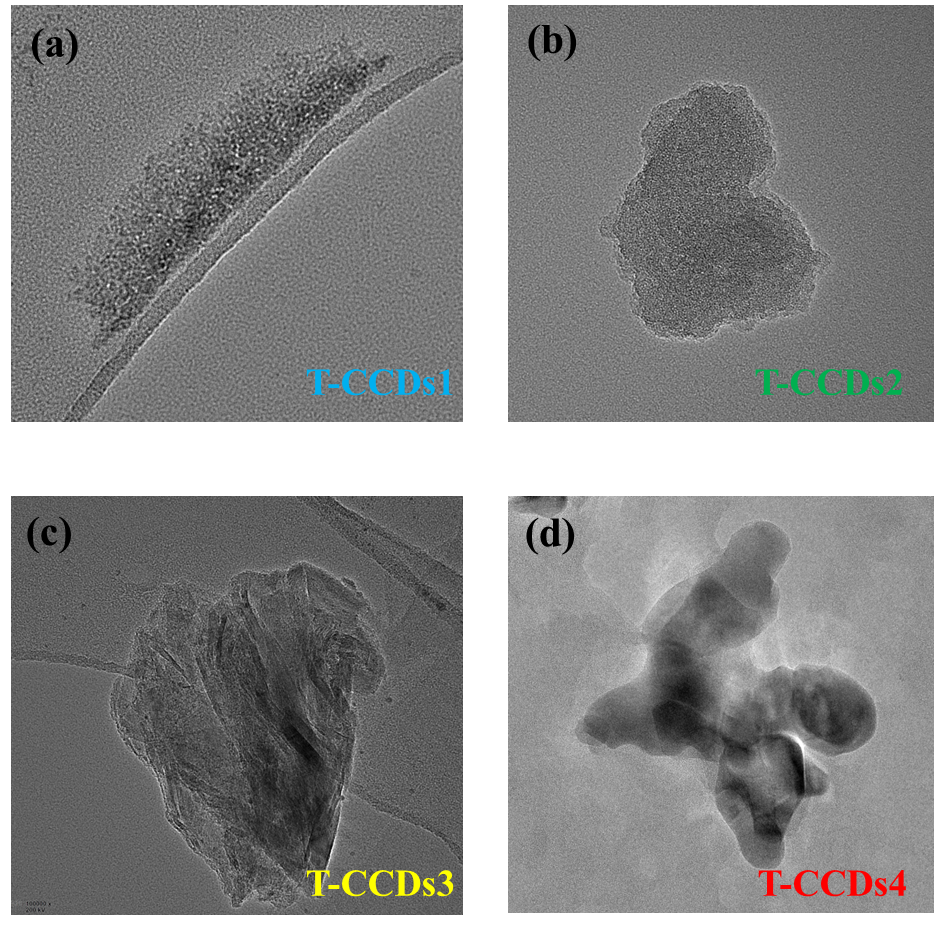
**

Figure S3. TEM image of the T-CCDs1-4 aggregated in water.


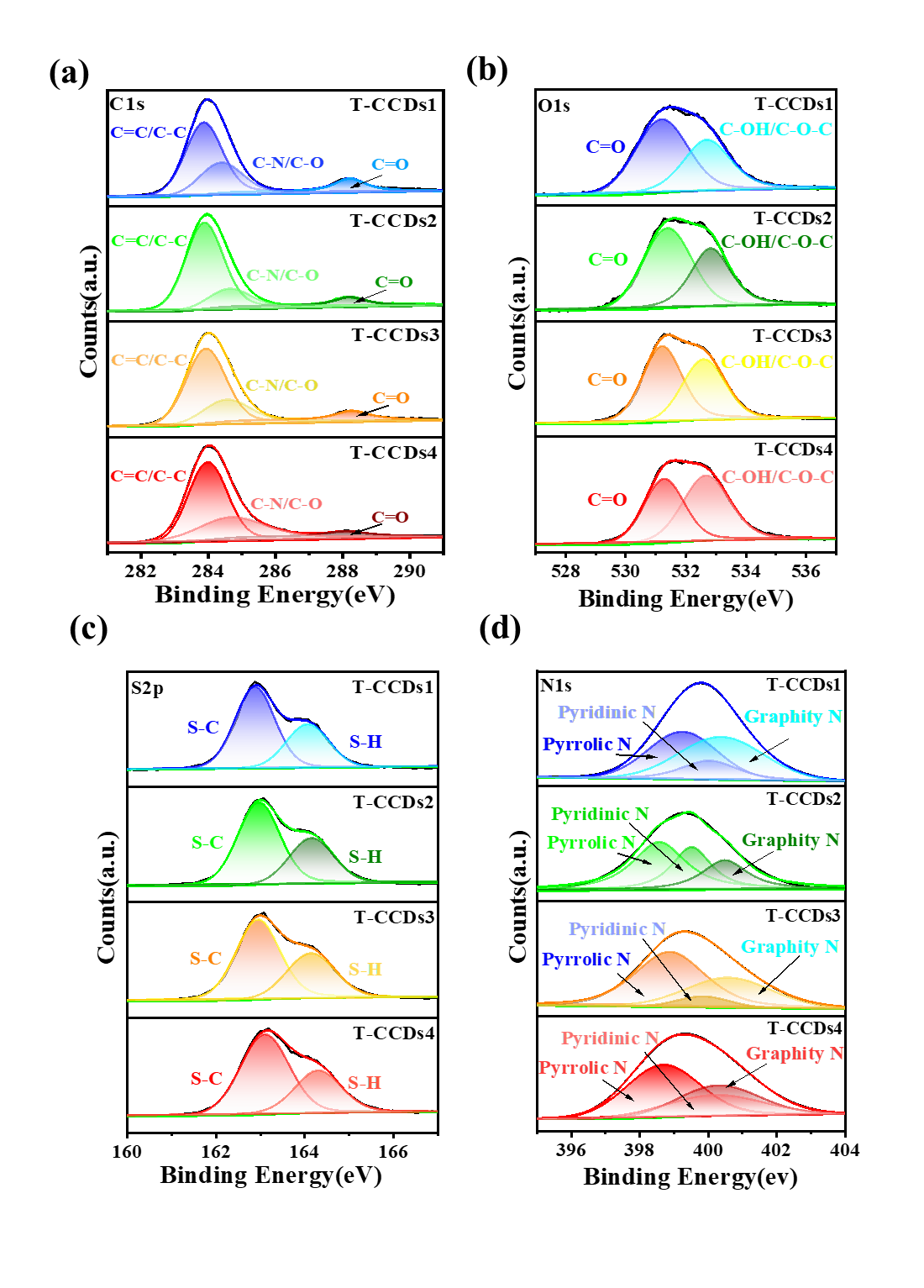


Figure S4. (a-d) high-resolution XPS spectra of T-CCDs1-4.

**Table S1.** XPS spectra of T-CCDs1-4.

| **/** | **Group** | **T-CCDs1** | **T-CCDs2** | **T-CCDs3** | **T-CCDs4** |
| --- | --- | --- | --- | --- | --- |
| **C 1s** | C=C/C-C | 13% | 9% | 11% | 7% |
|  | C-N/C-O | 27% | 16% | 22% | 31% |
|  | C=O | 61% | 75% | 67% | 62% |
| **N 1s** | Pyrrolic N | 44% | 26% | 35% | 31% |
|  | Pyridinic N | 15% | 34% | 7% | 6% |
|  | Graphitic N | 41% | 52% | 63% | 63% |
| **O 1s** | C=O | 42% | 42% | 45% | 55% |
|  | C-OH/C-O-C | 60% | 60% | 55% | 46% |
| **S 2p** | S-C | 35% | 35% | 36% | 35% |
|  | S-H | 67% | 67% | 65% | 29% |


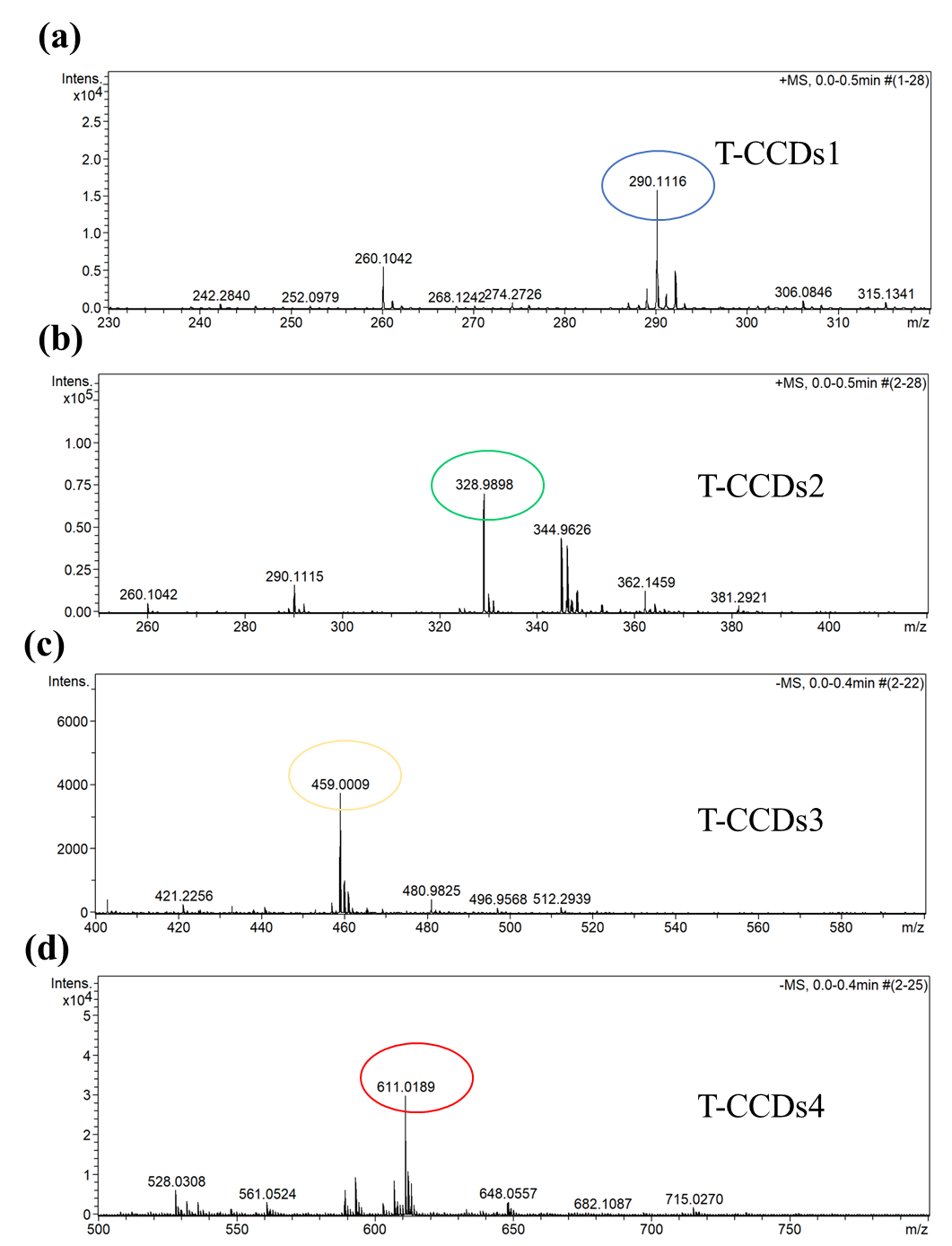


**Figure S5**. MS spectra of T-CCDs1-4 powder.

**
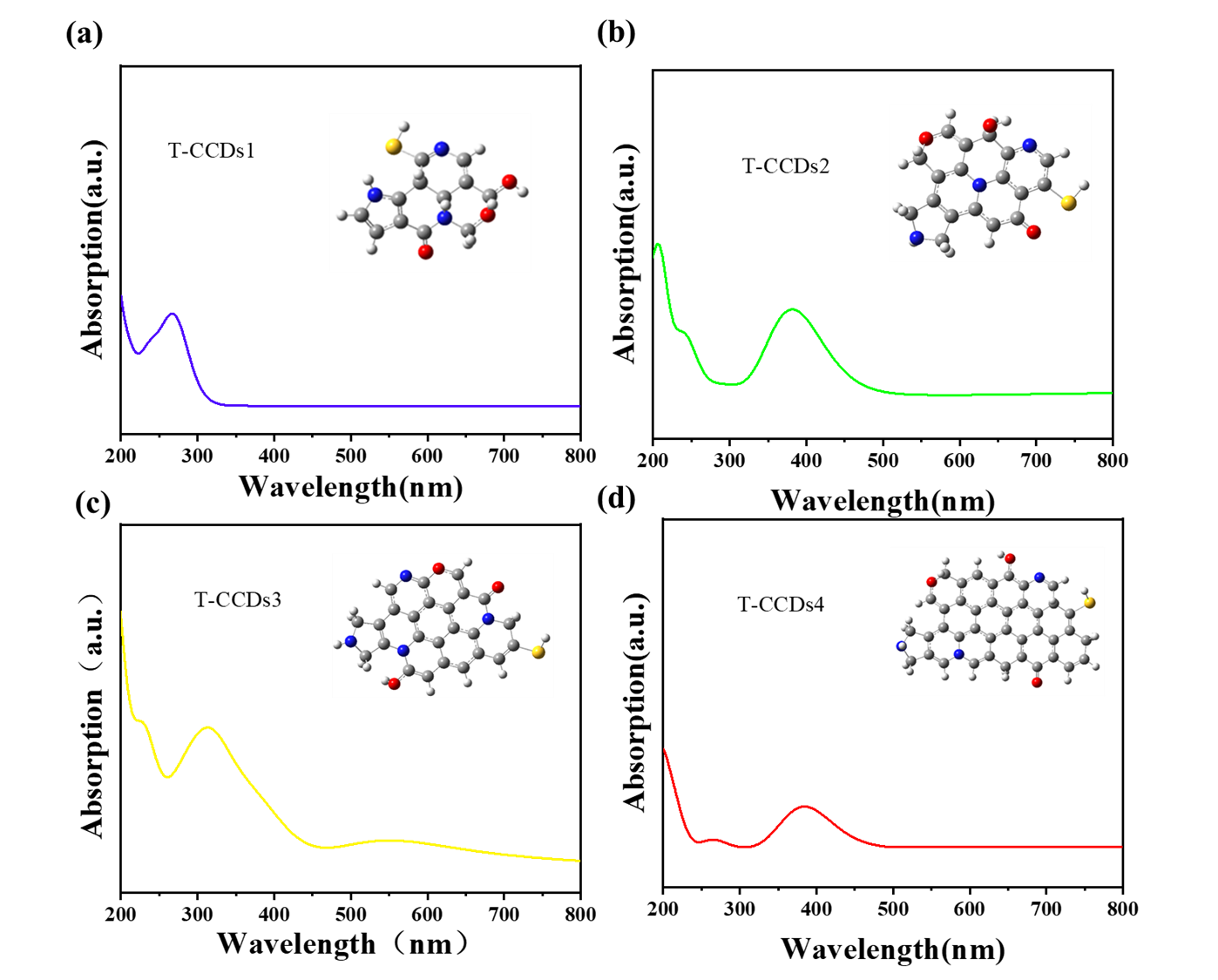
**

**Figure S6.** (a-d) The calculated UV-vis absorption spectra of T-CCDs1-4.

**
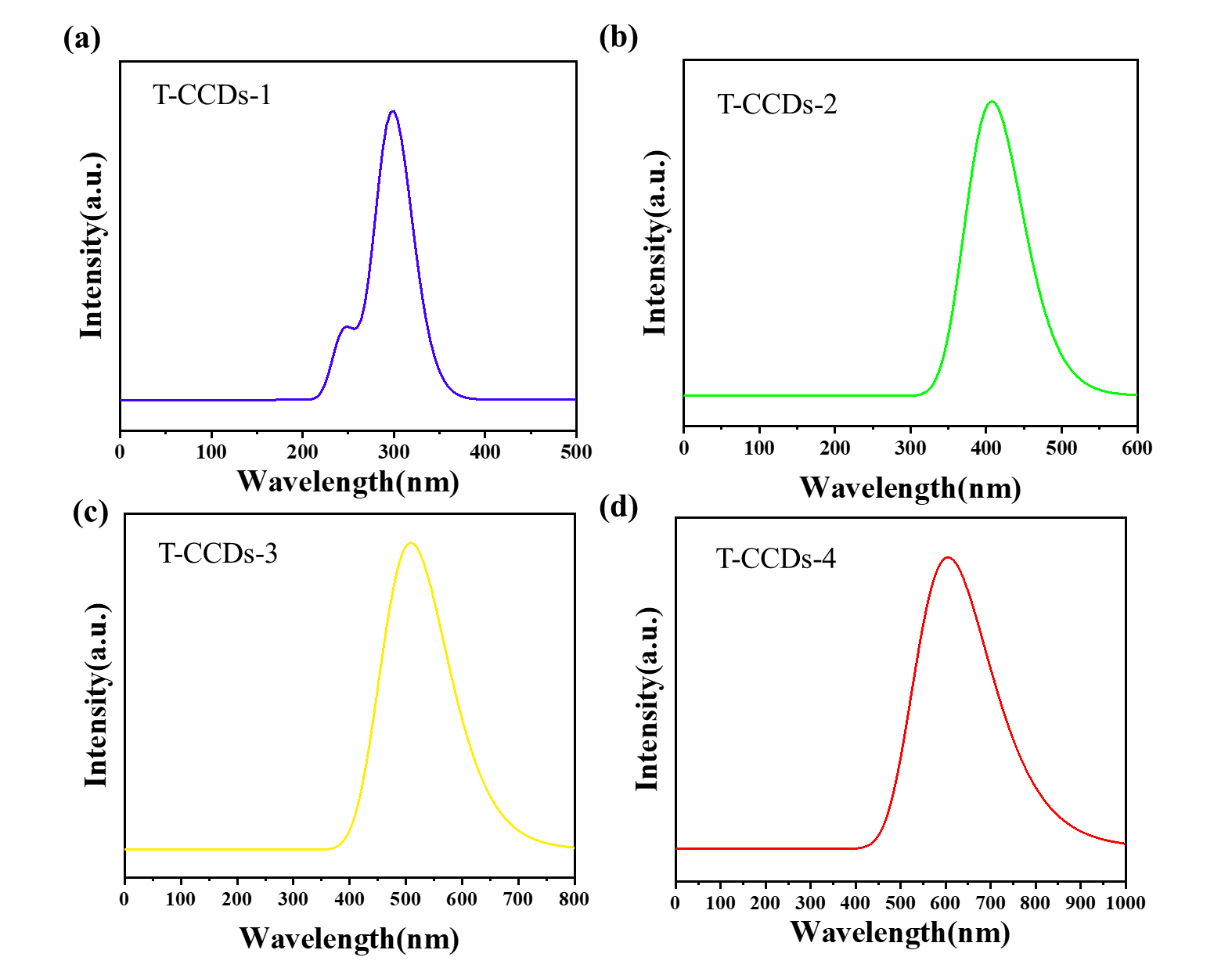
**

**Figure S7.** (a-d) The calculated fluorescence emission spectra of T-CCDs1-4.


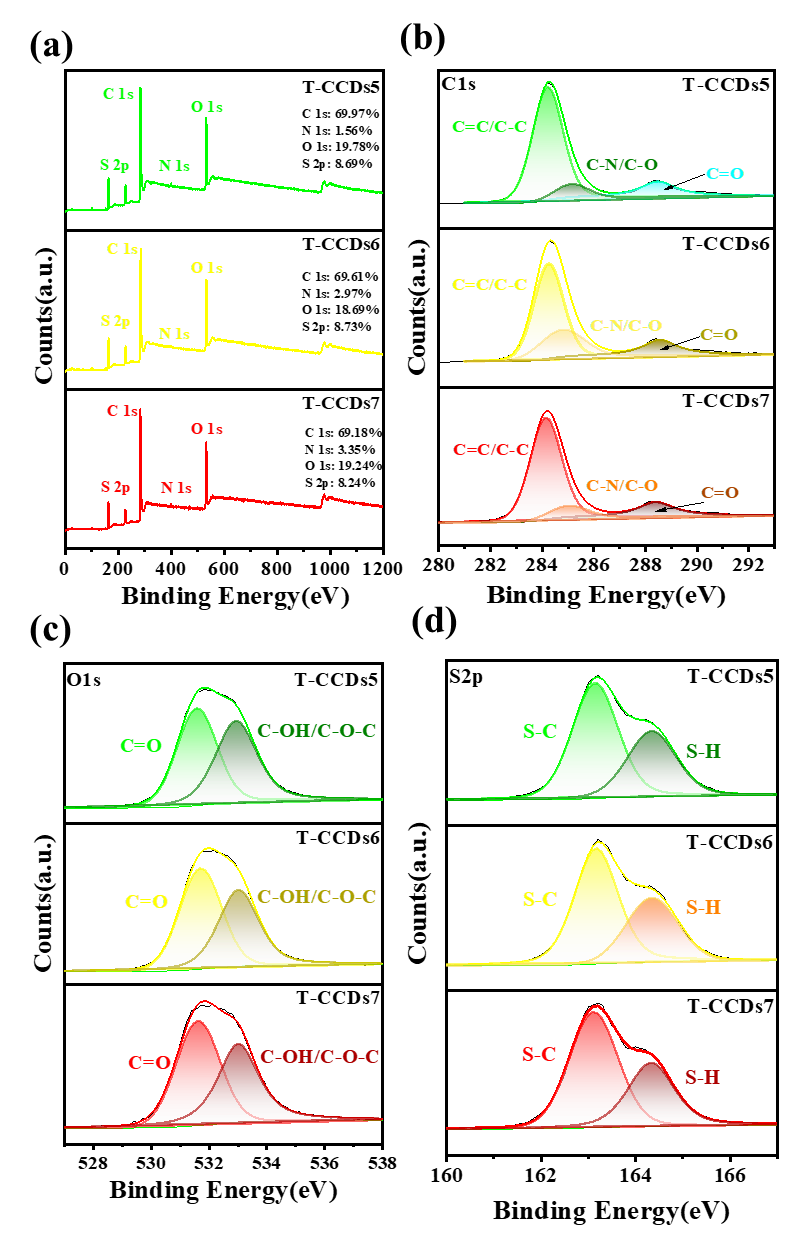


**Figure S8.** (a-d) high-resolution XPS spectra of T-CCDs5-7.

**Table S2** Elemental analysis of three kinds of T-CCDs5-7.

| **/** | **Group** | **T-CCDs5** | **T-CCDs6** | **T-CCDs7** |
| --- | --- | --- | --- | --- |
| **C 1s** | **C=C/C-C** | **16.8%** | **16.7%** | **17.2%** |
|  | **C-N/C-O** | **9.4%** | **24.5%** | **10.0%** |
|  | **C=O** | **75.7%** | **60.0%** | **74.7%** |
| **N 1s** | **Pyrrolic N** | **11.9%** | **9.8%** | **69.3%** |
|  | **Pyridinic N** | **77.1%** | **68.3%** | **6.7%** |
|  | **Graphitic N** | **11.7%** | **22.0%** | **24.4%** |
| **O 1s** | **C=O** | **51.4%** | **47.5%** | **49.5%** |
|  | **C-OH/C-O-C** | **52.4%** | **55.4%** | **54.7%** |
| **S 2p** | **S-C** | **38.6%** | **37.4%** | **38.2%** |
|  | **S-H** | **64.2%** | **65.1%** | **65.0%** |

**Figure S9.** FT-IR spectra of T-CCDs5-7.


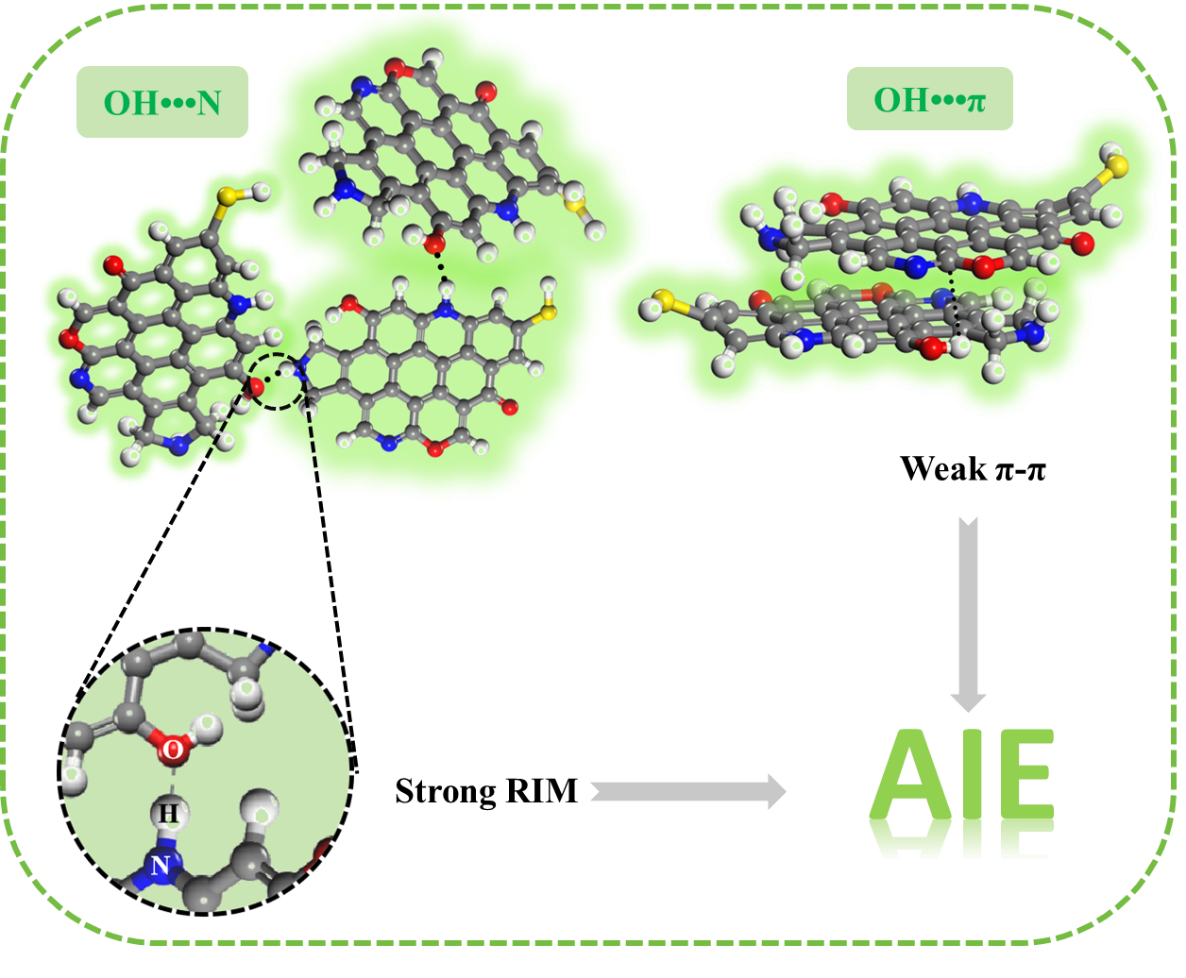


**Figure S10**. Schematic of hydrogen bonding interaction and AIE mechanism


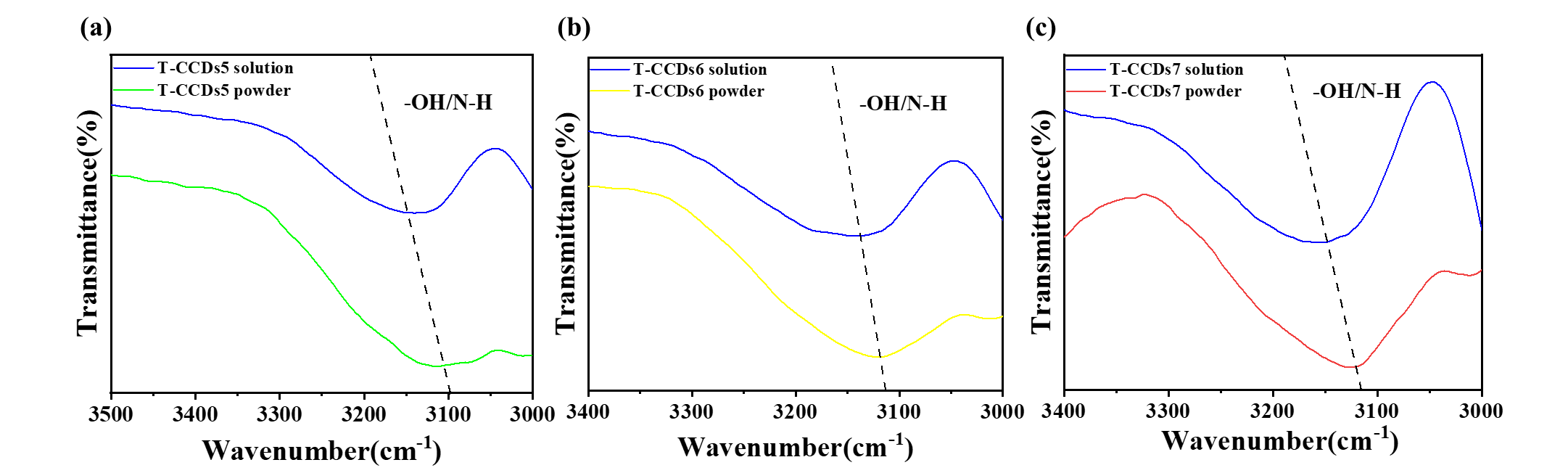


**Figure S11.** FT-IR spectra of T-CCDs5-7 powder and solution.


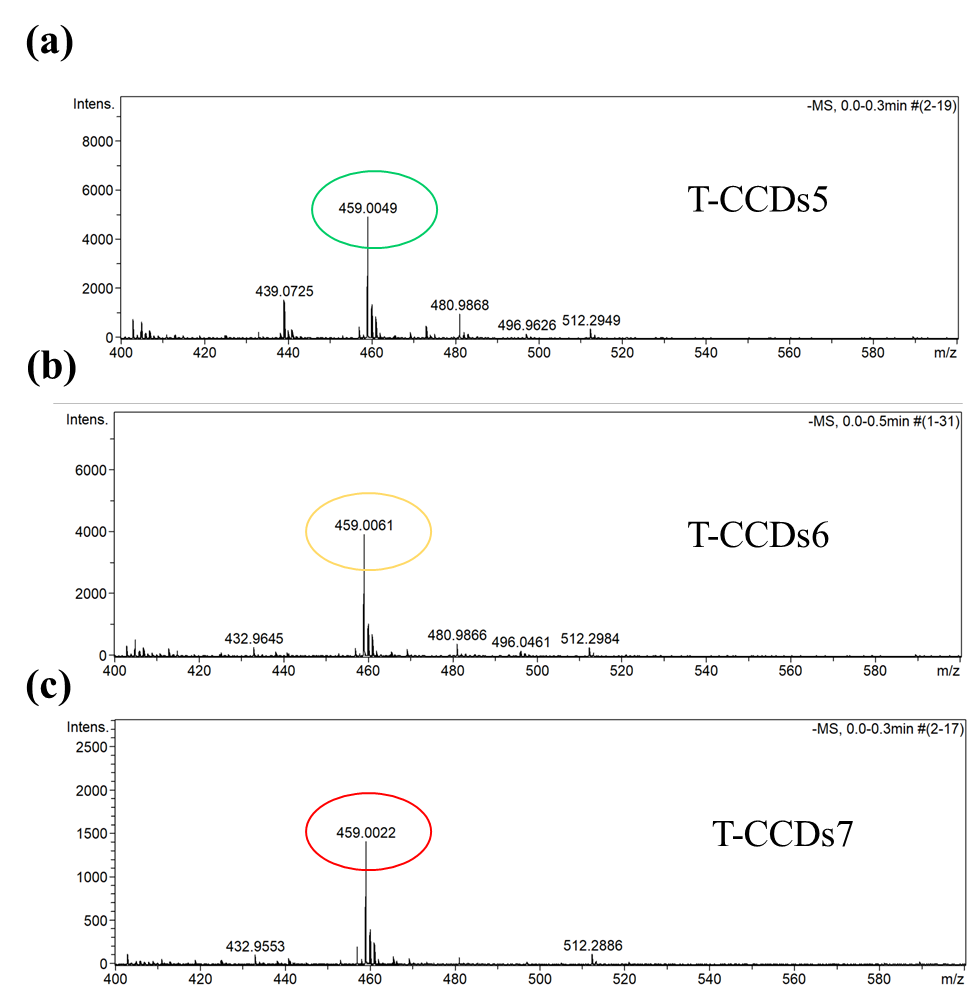


**Figure S12**. MS spectra of T-CCDs5-7 powder.

**
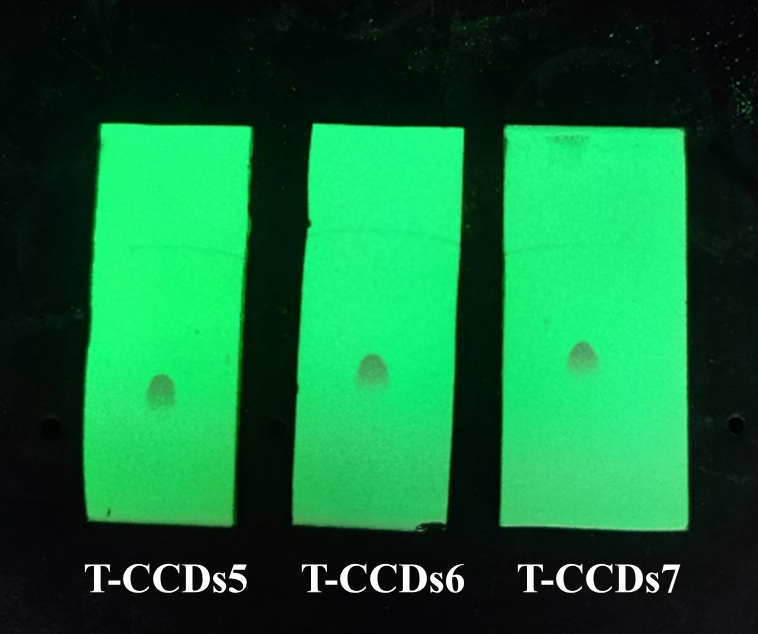
**

**Figure S13**. Thin-layer plate of synthesized T-CCDs5-7.

**Figure S14**. Raman spectra of T-CCDs5-7.


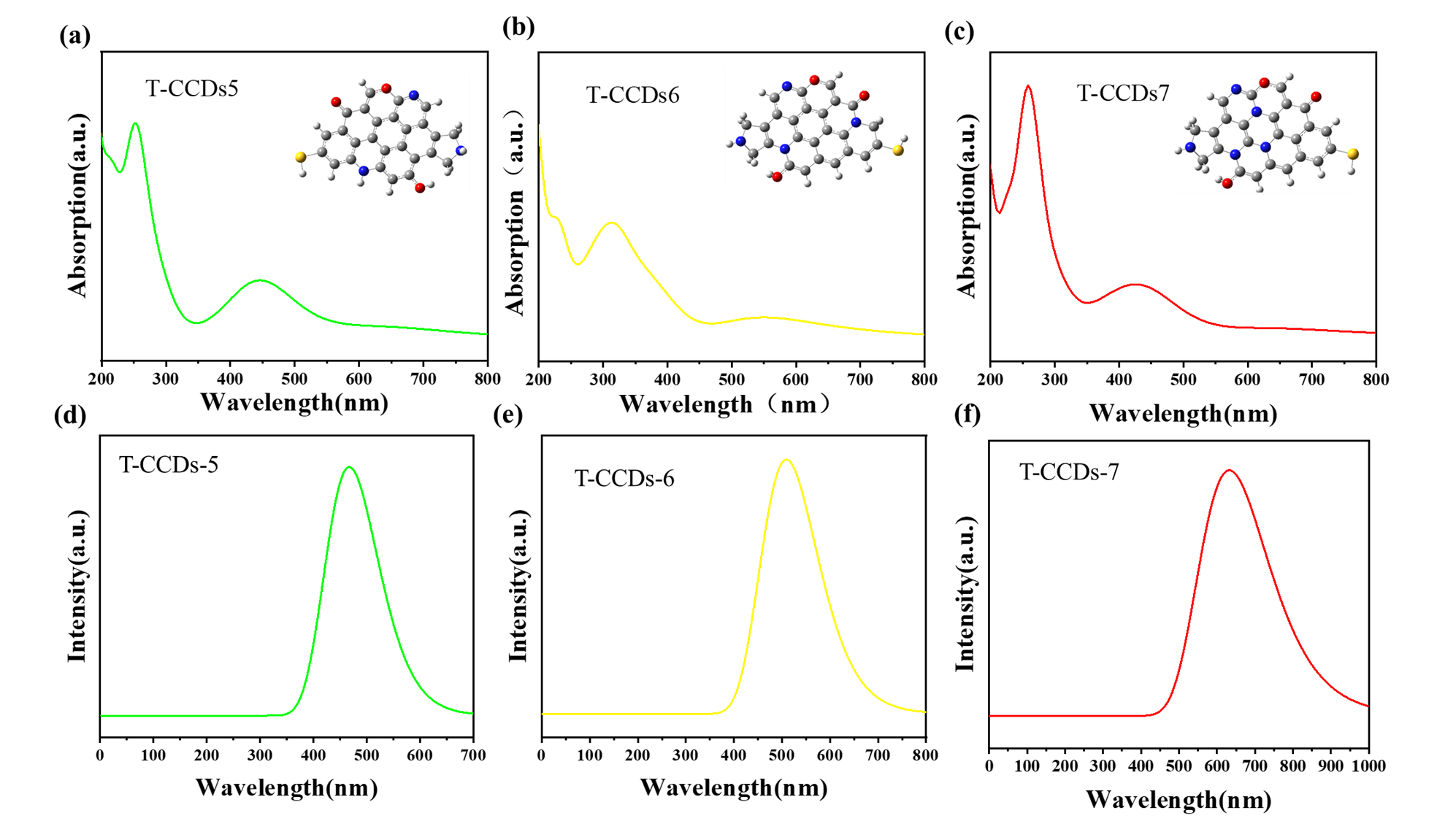


**Figure S15.** (a-c) The calculated UV-vis absorption spectra of T-CCDs5-7;


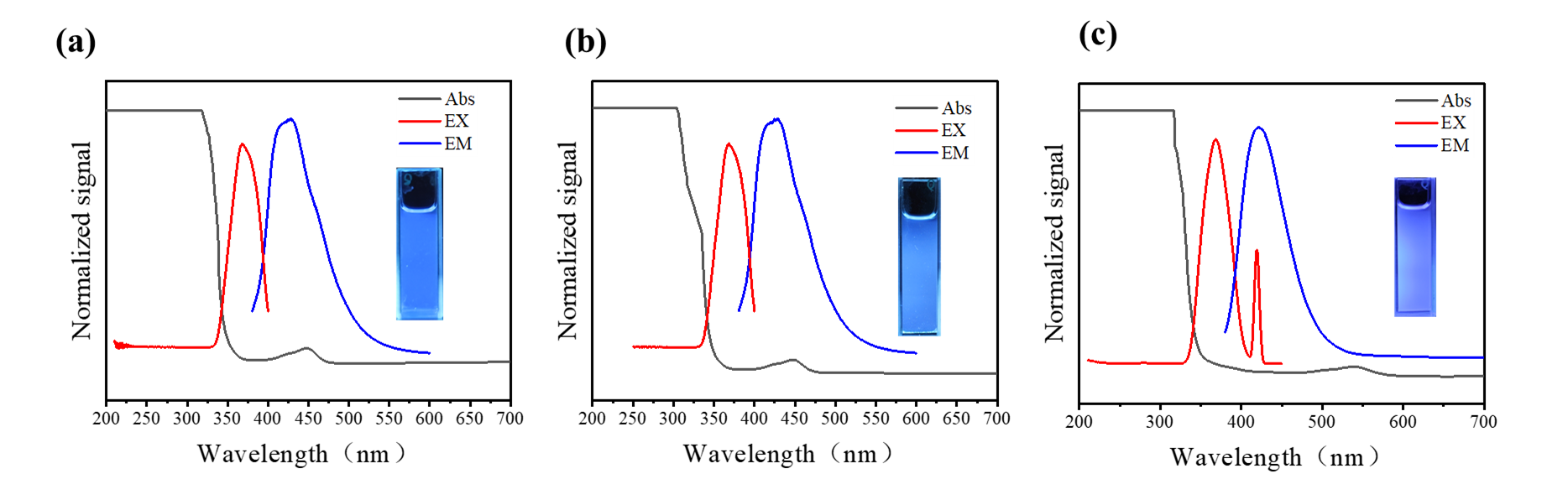


**Figure S16**. (a-c) UV-Vis absorption, FL excitation (Ex), and emission (Em) of as-prepared T-CCDs5-7 solution.

**Table S3.** Comparison of other CDs.

| No. | Scheme of relative CDs | Reaction condition | Wavelength range | Factors affecting the emissive color | State | Ref. |
| --- | --- | --- | --- | --- | --- | --- |
| 1 | 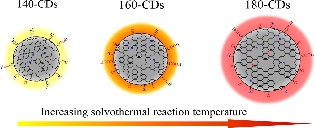 | Temperature | 466~650 nm | sp^2^-conjugated domains、surface-related state | Solid | [1] |
| 2 | 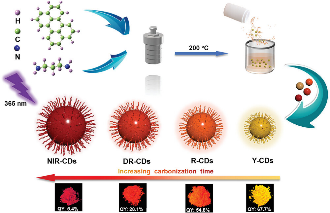 | Precursors and introduced acid reagents | 570~721 nm | Enlarged sp2 carbon crystal cluster | Solid | [2] |
| 3 | 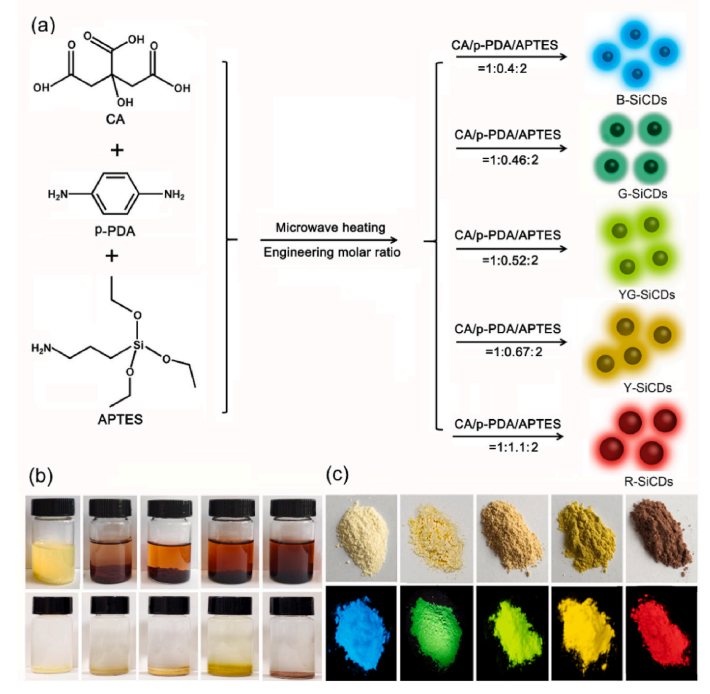 | precursor ratios | 438~633 nm | The size of sp2-conjugated domains and the content of graphitic nitrogen in carbon cores. | Solid | [3] |
| 4 | 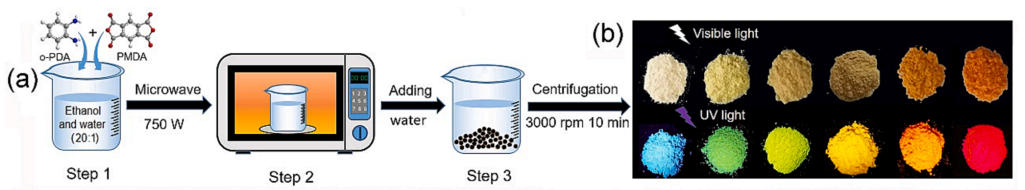 | Ratio of precursors and reaction time | 443~613 nm | The increasing amount of aromatic structures on the CD | Solid | [4] |
| 5 | 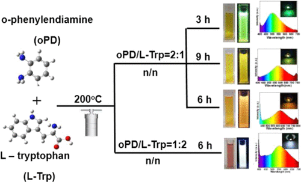 | Reaction time and precursor ratios | 535~602 nm | Extended sp^2^ domain or conjugated length and increasing amount of C=O groups and graphitic N | Solution | [5] |
| 6 | 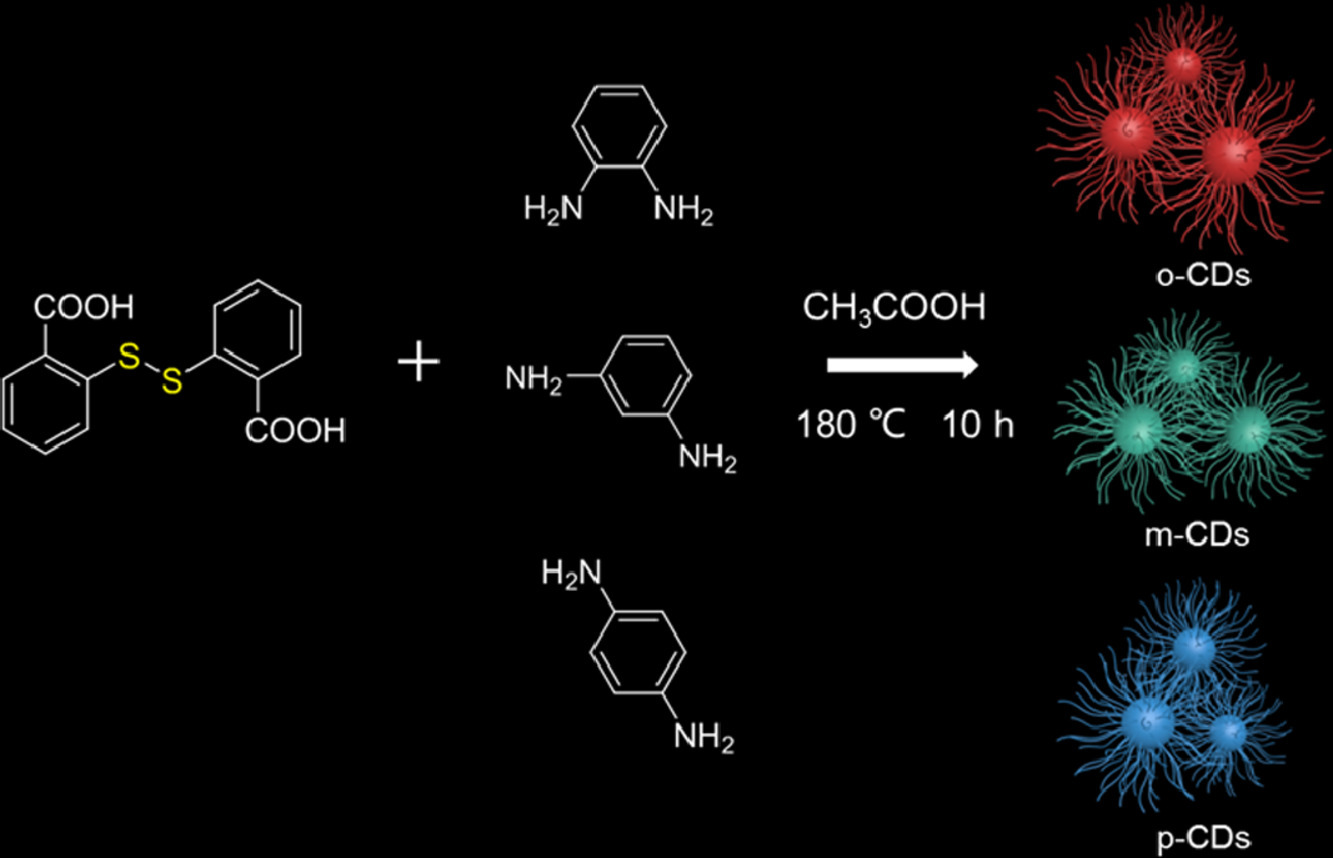 | Different precursor | 478~620 nm | The degree of graphitization and the increase of C=O content | Solid | [6] |
| 7 | 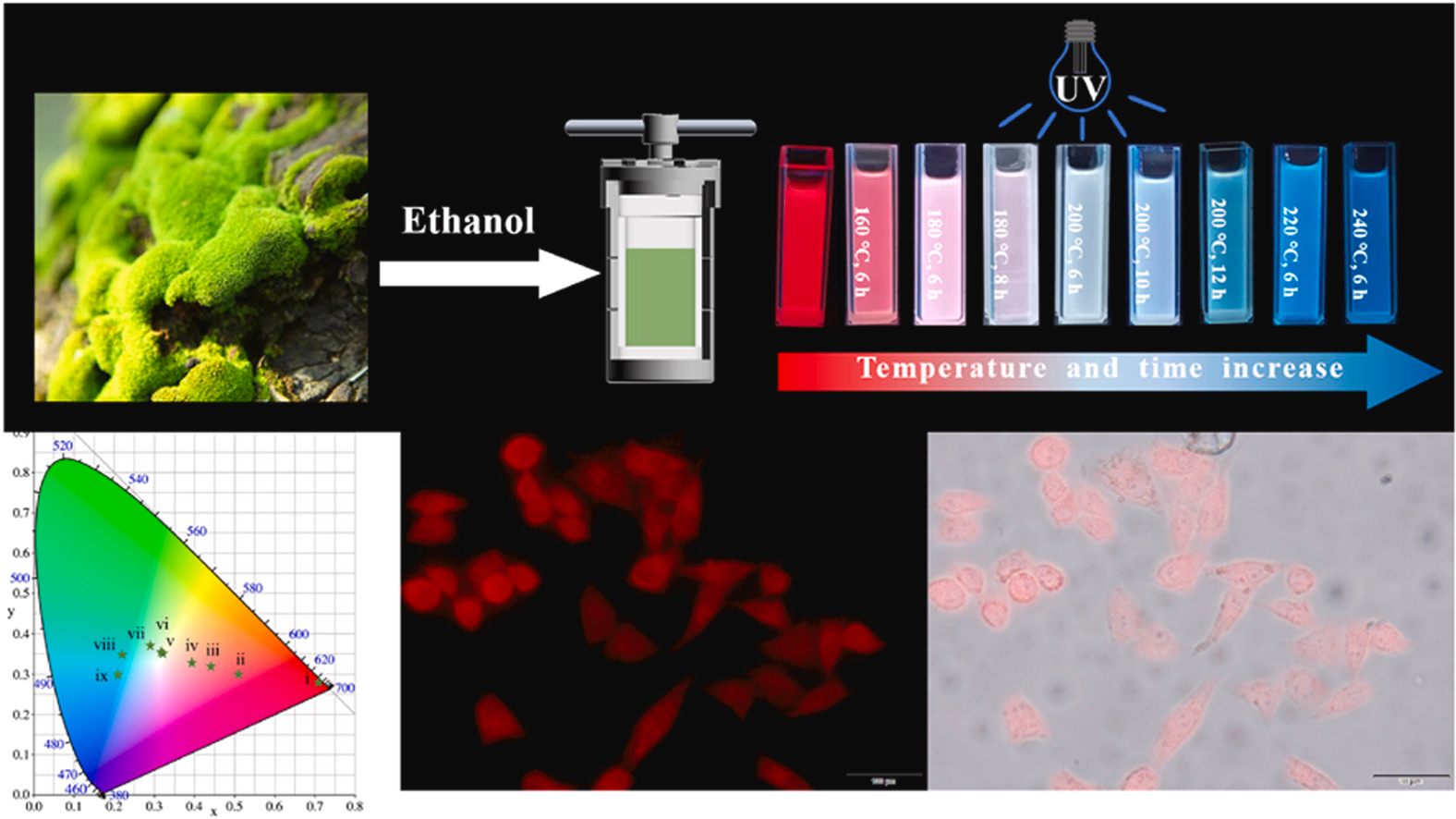 | Reaction temperature and time | 440~676 nm | control the ratio of chlorophyll as the red luminescent center to carbon nucleus as the blue luminescent center | Solution | [7] |
| 8 | 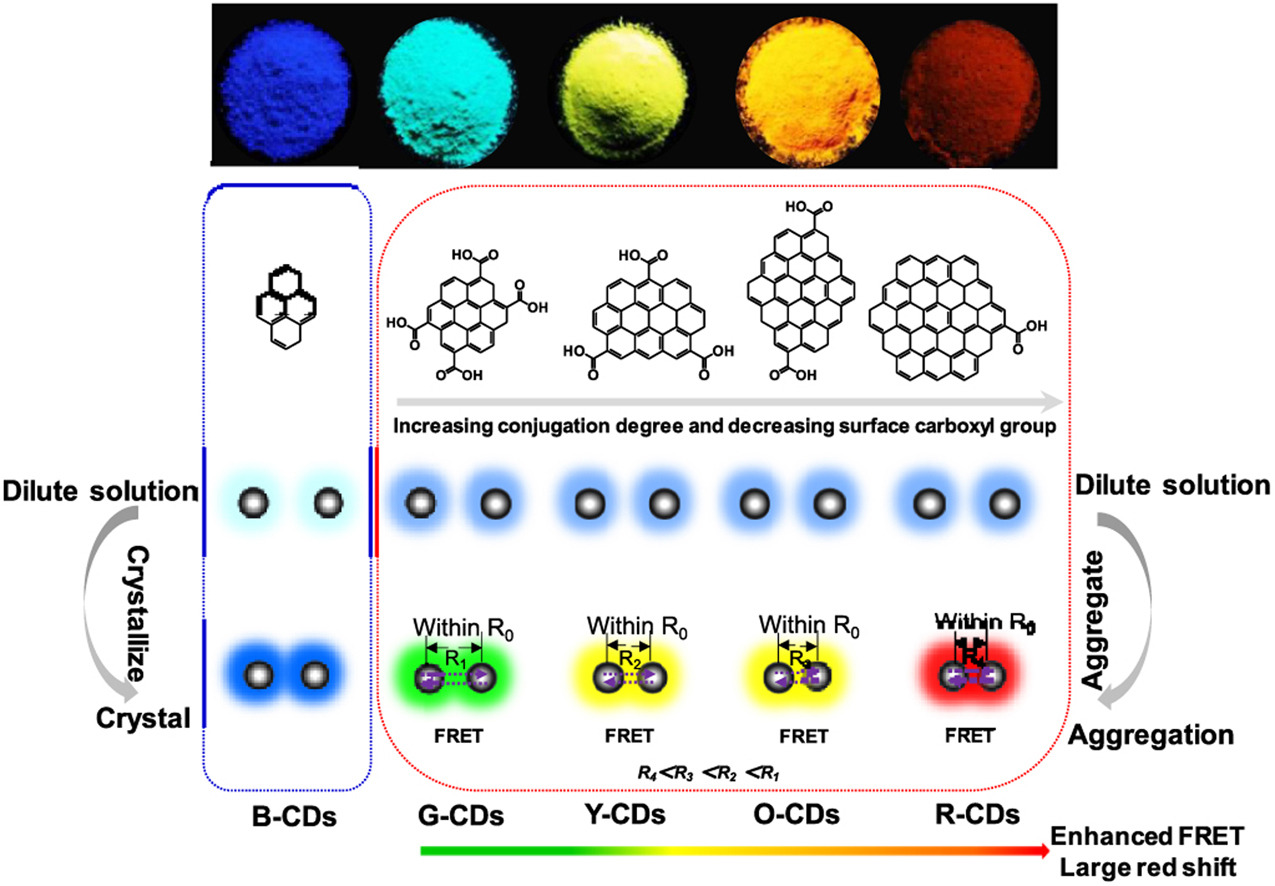 | Microwave power and precursor ratios | 445~643 nm | Increase size of conjugated sp2 domain and decreased carbonxy content synergistically | Solid | [8] |
| 9 | 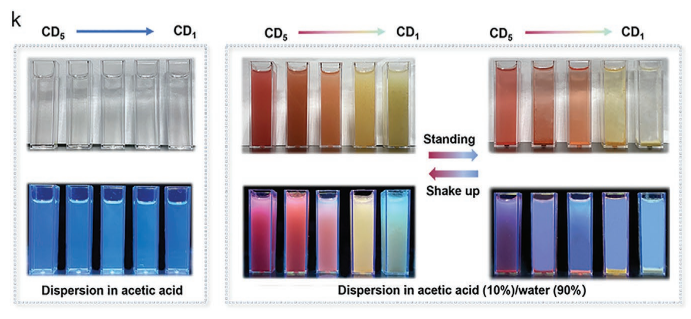 | Different precursor | 480~620 nm | the nitrogen content | Solid | [9] |
| 10 | 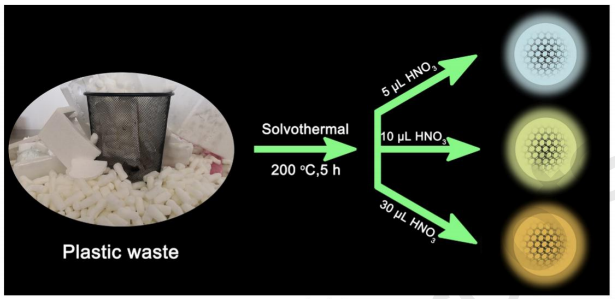 | Different precursor | 470~630 nm | The surface alkyl residues were unable to completely disperse the powdered CDs | Solid | [10] |

[1] B. Zhang, B. Wang, G. Xing, Z. Tang, S. Qu, *Chinese Chemical Letters* **2024**, *35* (9), https://doi.org/10.1016/j.cclet.2023.109358.

[2] B. Xu, J. Li, J. Zhang, H. Ning, X. Fang, J. Shen, H. Zhou, T. Jiang, Z. Gao, X. Meng, Z. Wang, *Adv Sci (Weinh)* **2023**, *10* (4), e2205788, https://doi.org/10.1002/advs.202205788.

[3] G. Hu, Y. Wang, S. Zhang, H. Ding, Z. Zhou, J. Wei, X. Li, H. Xiong, *Carbon* **2023**, *203*, 1, https://doi.org/10.1016/j.carbon.2022.11.048.

[4] H. Ding, R. Zhao, Z.-H. Zhang, J.-J. Yang, Z. Wang, L.-L. Xiao, X.-H. Li, X.-J. He, H.-M. Xiong, *Chemical Engineering Journal* **2023**, *476*, https://doi.org/10.1016/j.cej.2023.146405.

[5] J. Shen, X. Zheng, L. Lin, H. Xu, G. Xu, *ACS Applied Nano Materials* **2023**, *6* (4), 2478, https://doi.org/10.1021/acsanm.2c04764.

[6] X. Xu, L. Mo, W. Li, Y. Li, B. Lei, X. Zhang, J. Zhuang, C. Hu, Y. Liu, *Chinese Chemical Letters* **2021**, *32* (12), 3927, https://doi.org/10.1016/j.cclet.2021.05.056.

[7] F. Qin, J. Bai, P. He, X. Wang, S. Wu, X. Yu, Y. Yao, L. Ren, *Materials Today Chemistry* **2023**, *34*, 101816, https://doi.org/10.1016/j.mtchem.2023.101816.

[8] J. Wang, J. Zheng, Y. Yang, X. Liu, J. Qiu, Y. Tian, *Carbon* **2022**, *190*, 22, https://doi.org/10.1016/j.carbon.2022.01.001.

[9] X. Xu, L. Mo, Y. Li, X. Pan, G. Hu, B. Lei, X. Zhang, M. Zheng, J. Zhuang, Y. Liu, C. Hu, *Adv Mater* **2021**, *33* (49), e2104872, https://doi.org/10.1002/adma.202104872.

[10] H. Song, X. Liu, B. Wang, Z. Tang, S. Lu, *Sci Bull (Beijing)* **2019**, *64* (23), 1788, https://doi.org/10.1016/j.scib.2019.10.006.

**Table S4.** Comparison of other CDs in anti-counterfeiting application

| No. | Anti-counterfeiting application | The basis of anti-counterfeiting realization | Anti-counterfeiting type | Security level | Ref. |
| --- | --- | --- | --- | --- | --- |
| 1 | 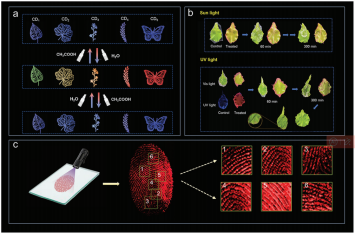 | AIE effect  Multi-color emission | dual-mode luminescence anti-counterfeiting | ☆☆☆ | [11] |
| 2 | 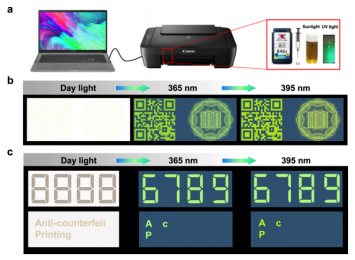 | invisibility under daylight | Inkjet printing | ☆☆ | [12] |
| 3 | 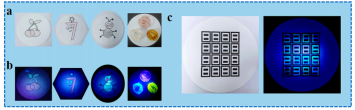 | invisibility under daylight  Multi-color emssion | Inkjet printing | ☆☆ | [13] |
| 4 | 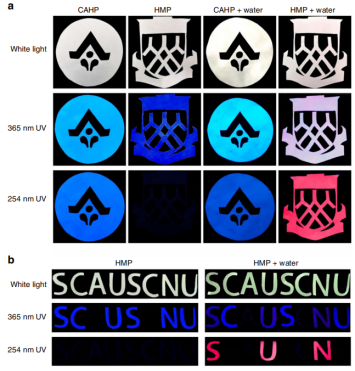 | AIE effect | Dual switch ink | ☆☆☆ | [14] |
| 5 | 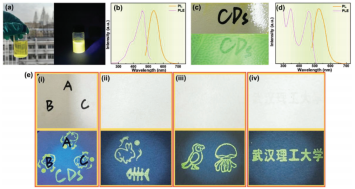 | Color change at different pH values | CDs combined with polyvinylpyrrolidone (PVP) inks | ☆☆☆ | [15] |
| 6 | 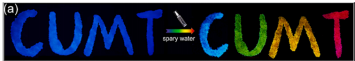 | AIE effect  Multi-color emssion | Dual switch ink | ☆☆☆ | [16] |
| 7 | 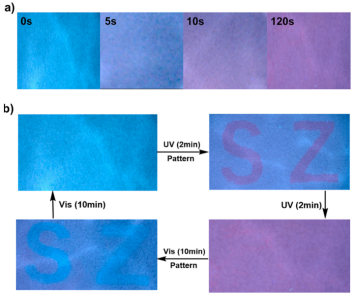 | photochromism and FRET | Inkjet printing | ☆☆☆☆ | [17] |
| 8 | 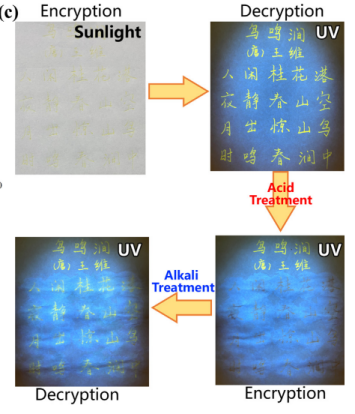 | Color change at different pH values | pH-sensitive fluorescent ink | ☆☆☆ | [18] |
| 9 | 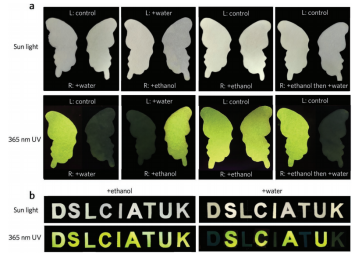 | Only AIE effect | Dual switch ink | ☆☆☆ | [19] |
| 10 | 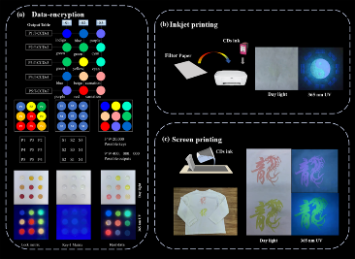 | AIE effect  Multi-color emission | Data-encryption  Inkjet printing  Screen printing | ☆☆☆☆☆ | **This work** |

[11] X. Xu, L. Mo, Y. Li, X. Pan, G. Hu, B. Lei, X. Zhang, M. Zheng, J. Zhuang, Y. Liu, C. Hu, *Adv Mater* **2021**, *33* (49), e2104872, https://doi.org/10.1002/adma.202104872.

[12] [1] L. Zhu, D. Shen, Q. Wang, K. H. Luo, *ACS Appl Mater Interfaces* **2021**, *13* (47), 56465, https://doi.org/10.1021/acsami.1c16679.

[13] W. Zhang, L. Li, M. Yan, J. Ma, J. Wang, C. Liu, Y. Bao, H. Jin, Q. Fan, *ACS Sustainable Chemistry & Engineering* **2023**, *11* (13), 5082, https://doi.org/10.1021/acssuschemeng.2c07045.

[14] H. Yang, Y. Liu, Z. Guo, B. Lei, J. Zhuang, X. Zhang, Z. Liu, C. Hu, *Nat Commun* **2019**, *10* (1), 1789, https://doi.org/10.1038/s41467-019-09830-6.

[15] M. Wu, J. Li, Y. Wu, X. Gong, M. Wu, *Small* **2023**, *19* (42), e2302764, https://doi.org/10.1002/smll.202302764.

[16] H. Ding, R. Zhao, Z.-H. Zhang, J.-J. Yang, Z. Wang, L.-L. Xiao, X.-H. Li, X.-J. He, H.-M. Xiong, *Chemical Engineering Journal* **2023**, *476*, https://doi.org/10.1016/j.cej.2023.146405.

[17] Y. Weng, Y. Hong, J. Deng, S. Cao, L. J. Fan, *J Colloid Interface Sci* **2024**, *655*, 622, https://doi.org/10.1016/j.jcis.2023.11.024.

[18] Z. Sun, W. Zhou, J. Luo, J. Fan, Z. C. Wu, H. Zhu, J. Huang, X. Zhang, *J Colloid Interface Sci* **2022**, *607* (Pt 1), 16, https://doi.org/10.1016/j.jcis.2021.08.188.

[19] Z. Wan, Y. Li, Y. Zhou, D. Peng, X. Zhang, J. Zhuang, B. Lei, Y. Liu, C. Hu, *Advanced Functional Materials* **2023**, *33* (11), 2207296, https://doi.org/10.1002/adfm.202207296.
